# Involvement of Protein Tyrosine Phosphatases BcPtpA and BcPtpB in Regulation of Vegetative Development, Virulence and Multi-Stress Tolerance in *Botrytis cinerea*

**DOI:** 10.1371/journal.pone.0061307

**Published:** 2013-04-09

**Authors:** Qianqian Yang, Fangwei Yu, Yanni Yin, Zhonghua Ma

**Affiliations:** Institute of Biotechnology, Zhejiang University, Hangzhou, China; Seoul National University, Republic of Korea

## Abstract

Tyrosine phosphorylation and dephosphorylation have emerged as fundamentally important mechanisms of signal transduction and regulation in eukaryotic cells, governing many processes, but little has been known about their functions in filamentous fungi. In this study, we deleted two putative protein tyrosine phosphatase (PTP) genes (*BcPTPA* and *BcPTPB*) in *Botrytis cinerea*, encoding the orthologs of *Saccharomyces cerevisiae* Ptp2 and Ptp3, respectively. Although BcPtpA and BcPtpB have opposite functions in conidiation, they are essential for sclerotial formation in *B. cinerea*. *BcPTPA* and *BcPTPB* deletion mutants ΔBcPtpA-10 and ΔBcPtpB-4 showed significantly increased sensitivity to osmotic and oxidative stresses, and to cell wall damaging agents. Inoculation tests showed that both mutants exhibited dramatically decreased virulence on tomato leaves, apples and grapes. In *S. cerevisiae*, it has been shown that Ptp2 and Ptp3 negatively regulate the high-osmolarity glycerol (HOG) pathway and the cell wall integrity (CWI) pathway. Although both BcPtpA and BcPtpB were able to inactive Hog1 and Mpk1 in *S. cerevisiae*, in contrast to *S. cerevisiae*, they positively regulate phosphorylation of BcSak1 (the homologue of Hog1) and BcBmp3 (the homologue of Mpk1) in *B. cinerea* under stress conditions. These results demonstrated that functions of PTPs in *B. cinerea* are different from those in *S. cerevisiae*, and BcPtpA and BcPtpB play important roles in regulation of vegetative development, virulence and in adaptation to oxidative, osmotic and cell-wall damage stresses in *B. cinerea*.

## Introduction

Protein phosphorylation and dephosphorylation executed by protein kinases and protein phosphatases are the most common mechanisms for regulating cellular processes. In eukaryotic cells, phosphorylation mainly occurs on three hydroxyl-containing amino acids, serine, threonine, and tyrosine. Accordingly, removal of the phosphate is catalyzed by protein Ser/Thr phosphatases, and tyrosine phosphatases (PTPs). In human, there are approximately 100 human PTP superfamily genes, compared to 90 human protein tyrosine kinase (PTK) genes, suggesting similar levels of complexity between the two families [1]. The levels of tyrosine phosphorylation in cells are determined by the balanced activity of PTKs and PTPs. Even the slightest tipping of this balance may result in cancer or abnormal cell death [2]. The regulation of PTPs is thus of major importance for governing many processes, including cell proliferation, cell cycle progression, metabolic homeostasis, transcriptional activation, neural transmission, differentiation and development, and aging [2].

Despite the overwhelming importance of PTPs in animals, studies on tyrosine phosphorylation have been relatively neglected in other eukaryotic cells. In plants, using several specific PTP inhibitors, MacRobbie demonstrates that PTP activities are essential for stomatal closure induced by four different factors including ABA, external calcium, darkness, and H_2_O_2_ [3]. In yeasts, the mitogen-activated protein kinases (MAPKs) have been shown to be inactivated by protein tyrosine phosphatases (PTPs) [4]–[7]. The *S. cerevisiae* MAPKs, Hog1 of the osmotic stress-activated high-osmolarity glycerol (HOG) pathway, Fus3 of the pheromone response pathway, and Mpk1 of the cell wall integrity pathway, are inactivated by two protein tyrosine phosphatases, Ptp2 and Ptp3 [8]. The two PTPs contain a catalytic domain of ∼400 residues sharing 57% similarity to each other [9]–[11]. Although Ptp2 and Ptp3 share similar functions in inactivating of MAPKs, Ptp2 is a more effective negative regulator of Hog1 than Ptp3 [6], [7], due to Ptp2 binds Hog1 more effectively than Ptp3 [6]. Similarly, both PTPs inactivate Mpk1, but Ptp2 is the more effective negative regulator [8]. In contrast, Ptp3 is a more effective negative regulator of Fus3 than Ptp2 [12].

*B. cinerea* is a necrotrophic plant pathogen causing gray mold in more than 200 plant species [13]. The pathogen is most destructive on mature or senescent tissues of dicotyledonous hosts. Global expenses of *Botrytis* control (including cultural measures, fungicide application, and biocontrol) easily surmount €1 billion/annum. The impacts of product loss occurring despite disease control, and the quality loss during the retail chain, are likely to be far higher [14]. In the last few years, the availability of the genome sequence and a variety of molecular tools together with its economic relevance have contributed to *B. cinerea* being one of the most extensively studied necrotrophic fungal pathogens.

A genome-wide search for PTPs in the filamentous fungi, including *B. cinerea*, *Neurospora crassa*, and *Magnaporthe oryzae*, revealed that all these genomes contain multiple putative PTP genes, suggesting the PTPs may be involved in key cellular processes as they are in yeast and human. Thus far, however, little is known about functions of these proteins in filamentous fungi. Thus, the aim of this study was to investigate the functions of PTPs genes *BcPTPA* and *BcPTPB* in *B. cinerea*.

## Results

### Sequence analysis of PTP genes in *B. cinerea*

According to amino acid similarity to *S. cerevisiae* Ptp2 and Ptp3, two putative PTP genes, named *BcPTPA* and *BcPTPB*, were retrieved from *B. cinerea* genome. The coding region of *BcPTPA* was 2,737-bp in length and was predicted to have two introns of 66-bp and 55-bp located after the 204^th^ and 1,791^th^ nucleotide, respectively. The existence of the introns was verified with reverse transcription PCR. The primer pair BcPtpA-F and BcPtpA-R (Table S1) generated a 2,616-bp and 2,737-bp fragment from cDNA and genomic DNA, respectively. Sequencing of the 2,616-bp product obtained from cDNA verified the predicted position and size of the introns. *BcPTPA* encodes an 872-amino acid protein, which shares 26% and 25% identity to *S. cerevisiae* Ptp2 and Ptp3, respectively.

The coding region of *BcPTPB* was 1,515-bp in length without intron. It was verified with reverse transcription PCR. The primer pair BcPtpB-F and BcPtpB-R (Table S1) generated the same 1,515-bp fragment from cDNA and genomic DNA. *BcPTPB* is predicted to encode a 505-amino acid protein. The conserved phosphatase catalytic domain of BcPtpB shares 24% and 30% identity to those of *S. cerevisiae* Ptp2 and Ptp3, respectively. In addition, BcPtpA and BcPtpB share 25% identity to each other.

### Deletion of *BcPTPA* and *BcPTPB*

To investigate the roles of BcPtpA and BcPtpB, we generated single gene deletion mutants of *BcPTPA* and *BcPTPB* using a homologous recombination strategy. For *BcPTPA*, three deletion mutants were identified from 98 hygromycin-resistant (HPH) transformants by PCR analysis with the primer pair BcPtpA-out-F and BcPtpA-out-R (Table S1). All three *BcPTPA* deletion mutants showed identical phenotypic characters. One ectopic mutant BcPtpA-5 which contains the intact wild-type gene and ectopic integration of the BcPtpA-upstream-HPH-BcPtpA-downstream cassette was also used in the following experiments. As shown in Figure S1C,D, Southern hybridization patterns confirmed that the two deletion mutants, ΔBcPtpA-2 and ΔBcPtpA-10 were the results from expected homologous recombination events at the *BcPTPA* locus and BcPtpA-5 is an ectopic mutant.

For *BcPTPB* gene, six deletion mutants were identified from 104 hygromycin-resistant transformants by PCR analysis with primer pair BcPtpB-F and BcPtpB-R (Table S1). Southern hybridization patterns confirmed that the *BcPTPB* deletion mutant ΔBcPtpB-4 was the result from expected homologous recombination events at the *BcPTPB* locus (Figure S1E).

### Involvement of BcPtpA and BcPtpB in the regulation of vegetative differentiation

**Figure 1:**
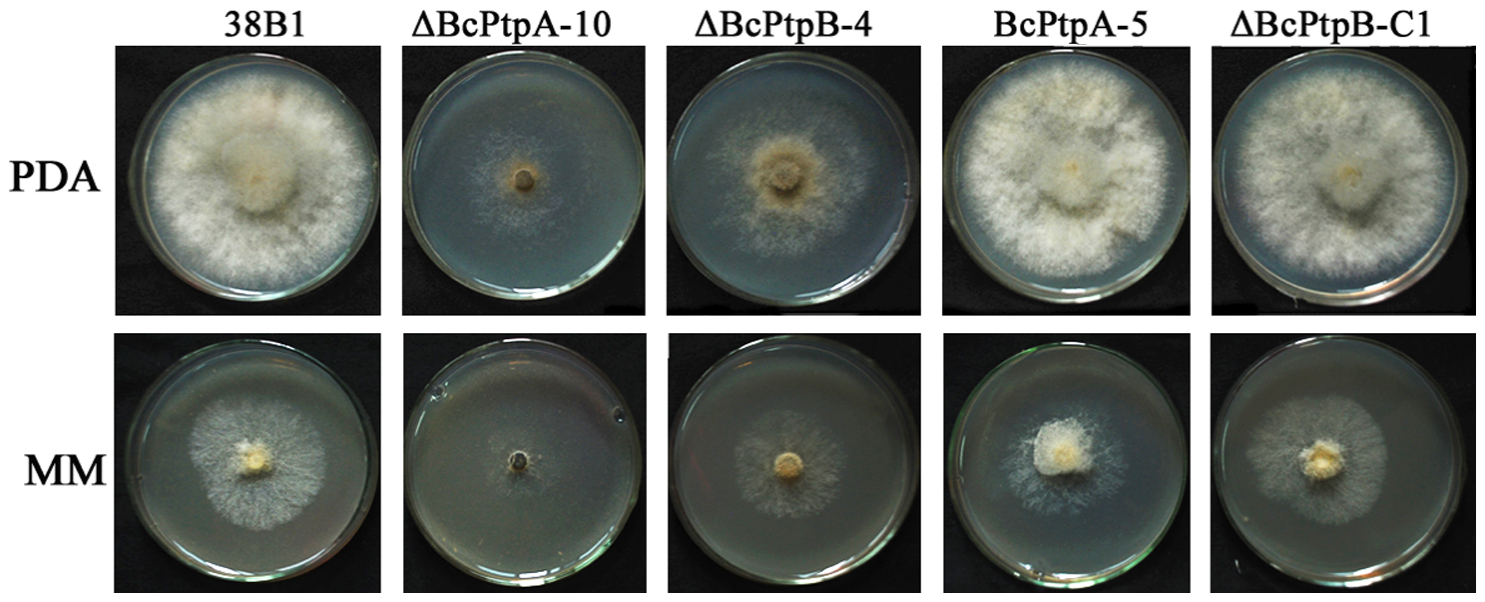
Colony morphology of the wild-type strain 38B1, *BcPTPA* deletion mutant ΔBcPtpA-10 and ectopic mutant BcPtpA-5, *BcPTPB* deletion mutant ΔBcPtpB-4, and its complemented strain ΔBcPtpB-C1 on potato dextrose agar (PDA) and minimal medium (MM). The pictures were taken after the plates were incubated at 25°C for 3 days.

ΔBcPtpA-10, to a lesser extent ΔBcPtpB-4, grew significantly slower than the wild-type progenitor 38B1 on either potato dextrose agar (PDA) or minimal medium (MM) (Figure 1). Microscopic examination of hyphae of ΔBcPtpA-10 and ΔBcPtpB-4 showed that compared to the wild-type strain, the mutants did not reveal remarkable changes in the hyphal branching, size and structure of hyphal cells (data not shown).

**Figure 2:**
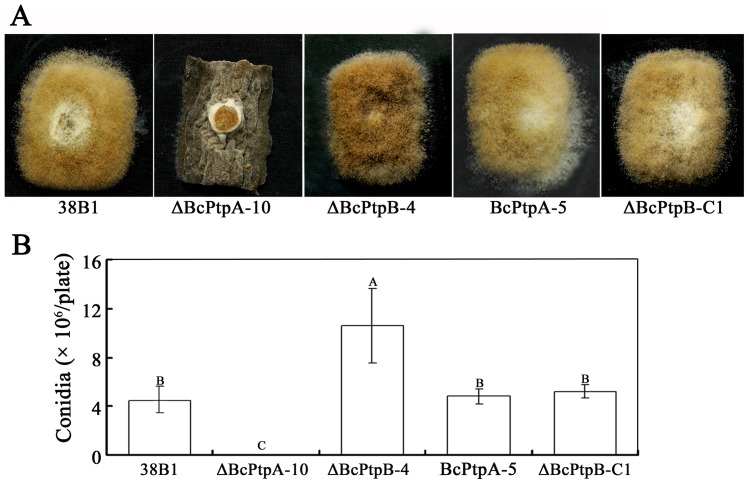
Comparisons in conidiation among 38B1, ΔBcPtpA-10, ΔBcPtpB-4, BcPtpA-5 and ΔBcPtpB-C1. (**A**) Colony morphology of the wild-type strain 38B1 and the mutants on sterilized cucumber fragments. The photos were taken after 10 days of incubation on sterilized cucumber fragments. (**B**) Quantification of conidia for each strain. The conidia of 38B1, ΔBcPtpA-10, ΔBcPtpB-4, BcPtpA-5 and ΔBcPtpB-C1 were washed off from each PDA plate after 10 days of incubation, and were counted under a microscope. Bars denote standard errors from three replications. Values on the bars followed by the same letter are not significantly different at *P = *0.05.

After incubated on PDA for 10 days, ΔBcPtpA-10 was unable to produce conidia. Since *B. cinerea* could produce more conidia on cucumber than on PDA medium, we also tested conidiation of the mutants on sterilized cucumber. After inoculation on autoclaved cucumber fragments for 10 days, the wild-type progenitor and the ectopic mutant BcPtpA-5 produced extensive aerial mycelia covered with a dense layer of conidia while ΔBcPtpA-10 produced only sparse aerial mycelia with few conidia (Figure 2). In contrast, ΔBcPtpB-4 produced significant more conidia than the wild-type progenitor 38B1 and complemented strain ΔBcPtpB-C1. The results indicate that BcPtpA and BcPtpB have opposite effects on conidiation in *B. cinerea*.

**Figure 3:**
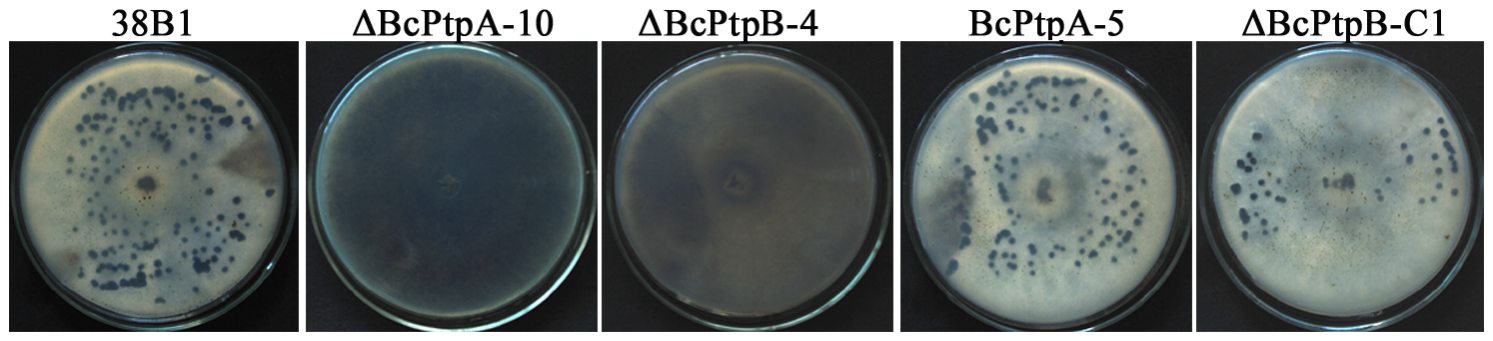
Impact of *BcPTPA* and *BcPTPB* deletion on sclerotial formation. The wild-type strain 38B1, ΔBcPtpA-10, ΔBcPtpB-4, BcPtpA-5 and ΔBcPtpB-C1 were incubated on PDA medium at 25°C for 4 weeks in darkness.

Because sclerotial formation within dying host tissues represents an important survival mechanism of *B. cinerea* in nature [15], we were interested in investigating effects of *BcPTPA* and *BcPTPB* deletion on sclerotial formation. After four weeks of incubation in the dark, ΔBcPtpA-10 and ΔBcPtpB-4 were unable to develop any sclerotia (Figure 3), indicating BcPtpA and BcPtpB are essential for sclerotial formation in *B. cinerea*.

### BcPtpA and BcPtpB regulate hypal melanization

**Figure 4:**
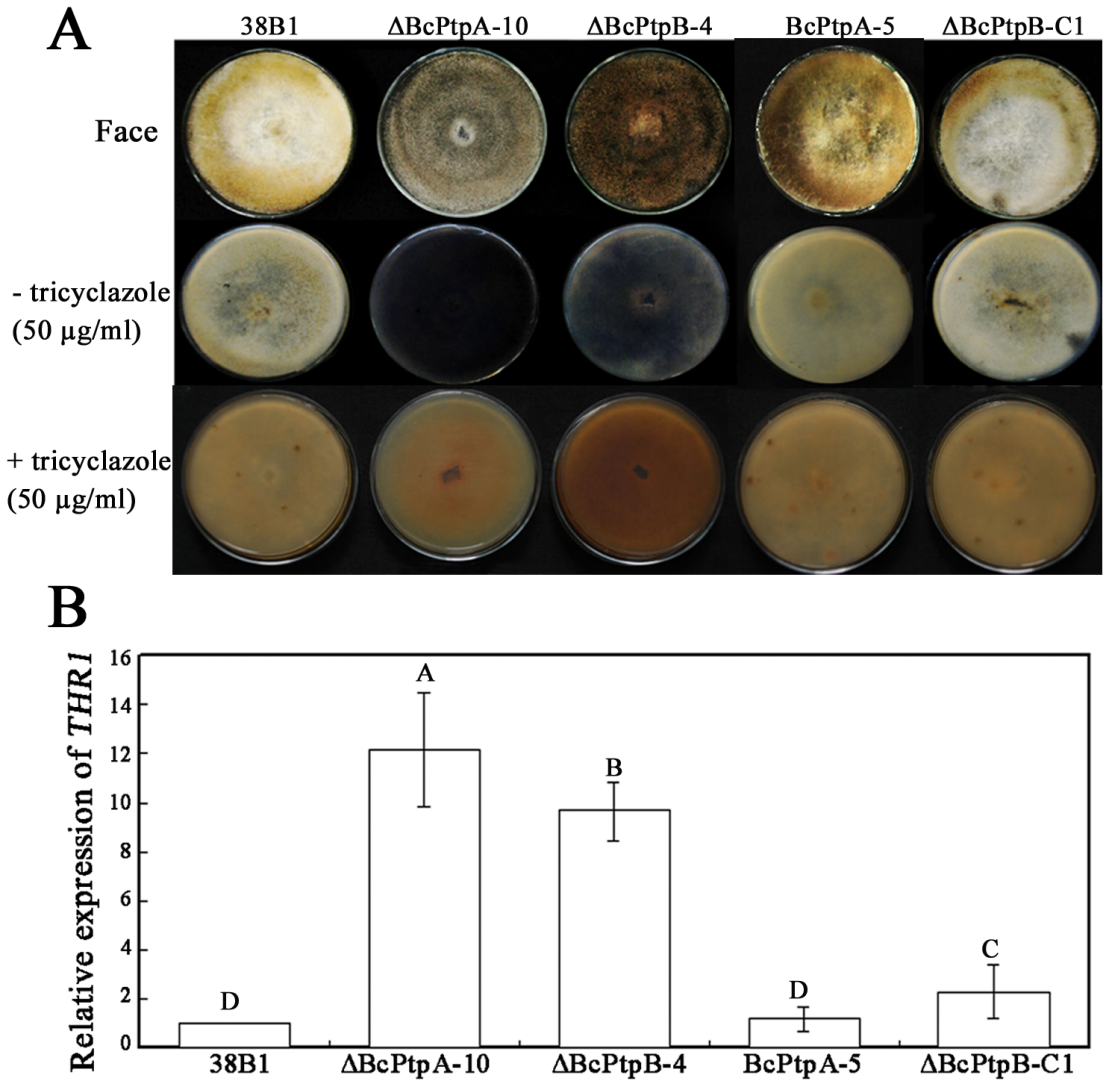
Involvement of *BcPTPA* and *BcPTPB* in the regulation of hypal melanization. (**A**) Comparisons of mycelial pigmentation among the wild-type strain 38B1, ΔBcPtpA-10, ΔBcPtpB-4, BcPtpA-5 and ΔBcPtpB-C1 after 9 days of incubation on PDA plates amended with or without 50 µg/ml tricyclazole. (**B**) Relative expression level of *THR1*, 1,3,8-trihydroxynaphthalene reductase gene, which is involved in melanin biosynthesis. Bars denote standard errors from three replications. Values on the bars followed by the same letter are not significantly different at *P = *0.05.

After incubation on PDA for 10 days, we found that lack of either *BcPTPA* or *BcPTPB* caused increased pigmentation (Figure 4A), indicating the mutants may produce more melanin. To test this hypothesis, ΔBcPtpA-10 and ΔBcPtpB-4 were incubated on PDA supplemented with 50 µg/ml tricyclazole, which is an inhibitor of fungal melanin biosynthesis [16], [17]. As shown in Figure 4A, both mutants were unable to produce the dark pigment on PDA amended with tricyclazole, verifying that the dark pigment produced by the mutants is melanin. These observations were further confirmed by significant over-expression of a melanin biosynthesis related gene, 1,3,8-trihydroxynaphthalene reductase gene (*THR1*) [18] in the mutants (Figure 4B). These results indicated that both BcPtpA and BcPtpB play a negative role in melanin biosynthesis in *B. cinerea*.

### Effects of *BcPTPA* and *BcPTPB* deletion on sensitivity of *B. cinerea* to fungicides, osmotic and oxidative stresses

**Figure 5:**
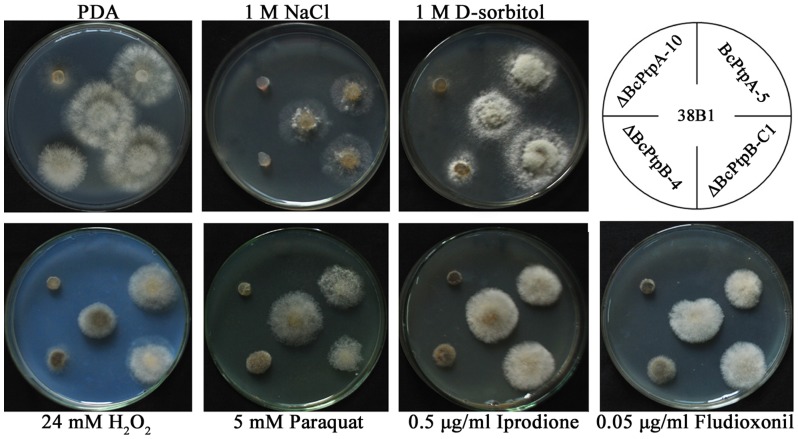
Sensitivity of 38B1, ΔBcPtpA-10, ΔBcPtpB-4, BcPtpA-5 and ΔBcPtpB-C1 to osmotic and oxidative stresses, and to fungicides. Comparisons were made on potato dextrose agar plates (PDA) amended with osmotic stress agents (NaCl and D-sorbitol), oxidative stress generators (H_2_O_2_ and paraquat), or each of iprodione and fludioxonil at the concentration described in the Figure. The pictures were taken after the plates were incubated at 25°C for 2 days.

It has been reported that osmotic and oxidative stresses, dicarboximide and phenylpyrrole fungicides could activate the HOG pathway in several fungal pathogens [19], we therefore tested the sensitivity of the mutants to various stresses. As shown in Figure 5, both ΔBcPtpA-10 and ΔBcPtpB-4 exhibited strongly increased sensitivity to osmotic stress mediated by NaCl at 1 M. Increased sensitivity of the mutants to osmotic stress was also observed on PDA amended with 1M D-sorbitol, but less pronounced. In addition, ΔBcPtpA-10 and ΔBcPtpB-4 also showed increased sensitivity to oxidative stresses generated by 24 mM H_2_O_2_ or 5 mM paraquat, and to the dicarboximide fungicide, iprodione, and the phenylpyrrole fungicide, fludioxonil. These results indicate that BcPtpA and BcPtpB may be involved in the HOG signal pathway in *B. cinerea*.

### Effects of *BcPTPA* and *BcPTPB* deletion on sensitivity of *B. cinerea* to cell wall-damaging agents and cell wall degrading enzymes

**Figure 6:**
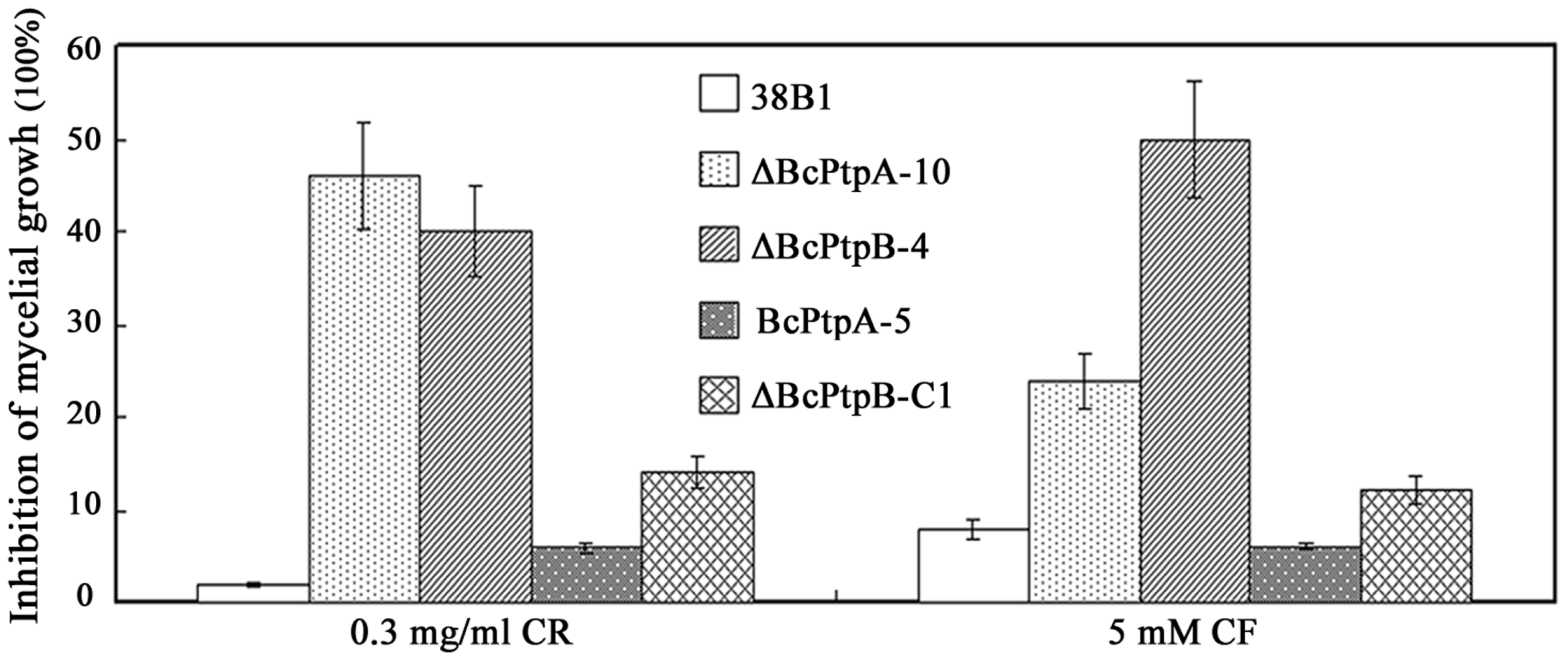
Sensitivity of 38B1, ΔBcPtpA-10, ΔBcPtpB-4, BcPtpA-5 and ΔBcPtpB-C1 to the cell wall-damaging agents Congo red (CR) and caffeine (CF). Bars denote standard errors from three replications.

**Figure 7:**
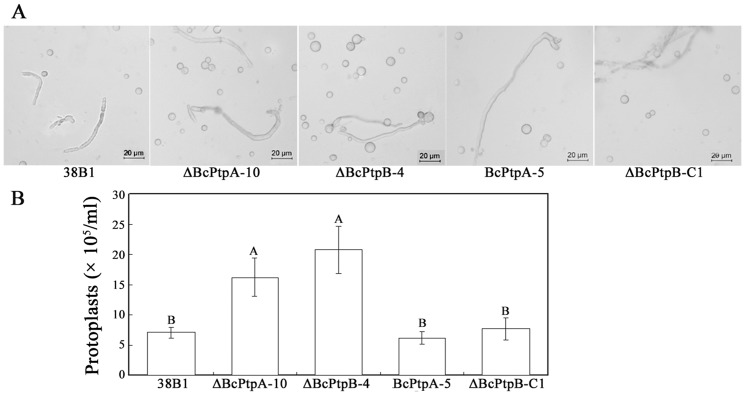
Sensitivity of 38B1, ΔBcPtpA-10, ΔBcPtpB-4, BcPtpA-5 and ΔBcPtpB-C1 to the cell-wall-degrading enzymes. (**A**) Fungal mycelia of each strain were cultivated in YEPD medium for 28 h, washed and incubated for 2 h in osmotically stabilized solution (0.6 M KCl) containing 0.25% Glucanex before microscopic examination. (**B**) Protoplasts were counted microscopically after filtration from the remaining mycelium. Bars denote standard errors from three replications. Values on the bars followed by the same letter are not significantly different at *P = *0.05.

In a previous study, Liu et al. found that the osmotic signal transduction cascade is associated with cell wall integrity (CWI) in *B. cinerea* [20]. Thus, we were interested in examining the sensitivity of ΔBcPtpA-10 and ΔBcPtpB-4 to cell wall-damaging agents including Congo red (0.3 mg/ml) and caffeine (5 mM). Interestingly, both ΔBcPtpA-10 and ΔBcPtpB-4 exhibited increased sensitivity to cell wall damaging agents (Figure 6). Consistently, we observed that both mutants revealed increased sensitivity to cell wall degrading enzymes. As shown in Figure 7, ΔBcPtpA-10 and ΔBcPtpB-4 produced significant more protoplasts than the wild-type strain after 0.3 g fresh hyphae of each strain were treated with 0.25% lysing enzymes (Glucanex; Sigma, USA) for 2 h.

### Effects of *BcPTPA* and *BcPTPB* deletion on intracellular glycerol accumulation

**Figure 8:**
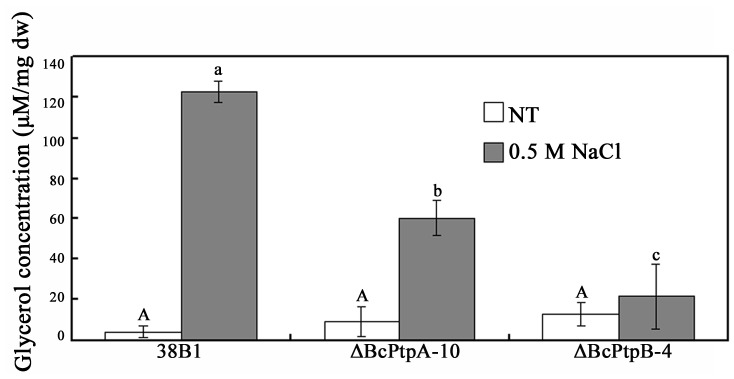
Comparisons in intracellular glycerol concentration among the wild-type strain 38B1, ΔBcPtpA-10, and ΔBcPtpB-4. Mycelia of each strain were treated with 0.5 M NaCl for 2 hours after grown in potato dextrose broth for 2 days. The cultures without treatment were used as the control (NT). Bars denote standard errors from three repeated experiments. Values on the bars followed by the same letter are not significantly different at *P = *0.05.

Since osmotic stress can induce glycerol accumulation in *S. cerevisiae* and *N. crassa* via the HOG pathway [21]-[23], and both ΔBcPtpA-10 and ΔBcPtpB-4 showed increased sensitivity to osmotic stresses, we therefore analyzed glycerol accumulation in mycelia of ΔBcPtpA-10 and ΔBcPtpB-4. As shown in Figure 8, in the absence of osmotic stress, very little glycerol was detected in the wild-type strain, and in ΔBcPtpA-10 and ΔBcPtpB-4 mutants. High salt treatment induced glycerol accumulation in all three strains, but the glycerol concentration in the wild type was significantly higher than that in each mutant (Figure 8).

### Regulation of BcSak1 and BcBmp3 phosphorylation by BcPtpA and BcPtpB

**Figure 9:**
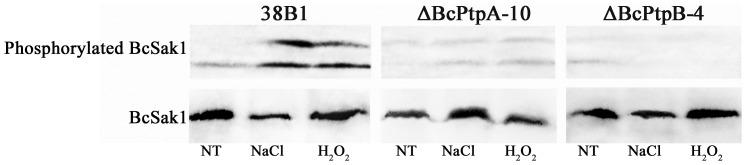
Phosphorylation levels of BcSak1 in 38B1, ΔBcPtpA-10, and ΔBcPtpB-4. Mycelia of each strain were treated with 0.5 M NaCl or 24 mM H_2_O_2_ for 2 hours after being grown in potato dextrose broth for 2 days. The cultures without any treatment were used as the control (NT). BcSak1 and phosphorylated BcSak1 proteins were detected using the yeast anti-Hog1p (C-terminal anti-Hog1) and phosphorylated p38 (Thr180/Tyr182) antibodies, respectively.

In *S. cerevisiae*, Ptp2 and Ptp3 negatively regulate the HOG pathway by dephosphorylating the Hog1 [6]–[8]. We therefore examined phosphorylation of BcSak1 (the ortholog of *S. cerevisiae* Hog1) in the mutants. In the wild type, BcSak1 phosphorylation was dramatically increased in response to osmotic stress (0.5 M NaCl) and oxidative stress (24 mM H_2_O_2_) (Figure 9). In ΔBcPtpA-10 and ΔBcPtpB-4, surprisingly, phosphorylation levels of BcSak1 remained very low (Figure 9), which indicates that in contrast to *S. cerevisiae*, neither BcPtpA nor BcPtpB is the negative regulator of BcSak1 in *B. cinerea* under stress conditions. These results are in agreement with the low levels of glycerol accumulation in ΔBcPtpA-10 and ΔBcPtpB-4.

**Figure 10:**
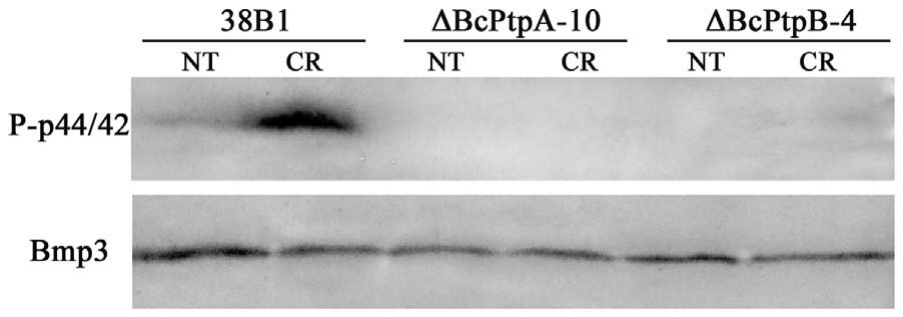
Phosphorylation levels of BcBmp3 in 38B1, ΔBcPtpA-10, ΔBcPtpB-4. Mycelia of each strain were treated with 0.3 mg/ml Congo red for 2 hours after being grown in potato dextrose broth for 2 days. The cultures without any treatment were used as the control (NT). BcBmp3 and phosphorylated BcBmp3 proteins were detected using the yeast anti-Mpk1 (yN-19) and phospho-p44/42 MAP kinase antibody (Cell Signaling) antibodies, respectively.

In *B. cinerea*, the HOG pathway also regulates phosphorylation status of Bmp3 (the ortholog of *S. cerevisiae* Mpk1 in CWI pathway) [20]. Therefore, we were also interested in examining phosphorylation levels of Bmp3 in ΔBcPtpA-10 and ΔBcPtpB-4. As shown in Figure 10, in the wild-type strain, BcBmp3 phosphorylation was drastically increased in response to 0.3 mg/ml Congo red treatment. In contrast, phosphorylation of BcBmp3 remained at a low level in ΔBcPtpA-10 and ΔBcPtpB-4, indicating that BcPtpA and BcPtpB are positive regulators of BcBmp3 in *B. cinerea* under stress conditions

### Requirement of BcPtpA and BcPtpB in full pathogenicity of *B. cinerea*

**Figure 11:**
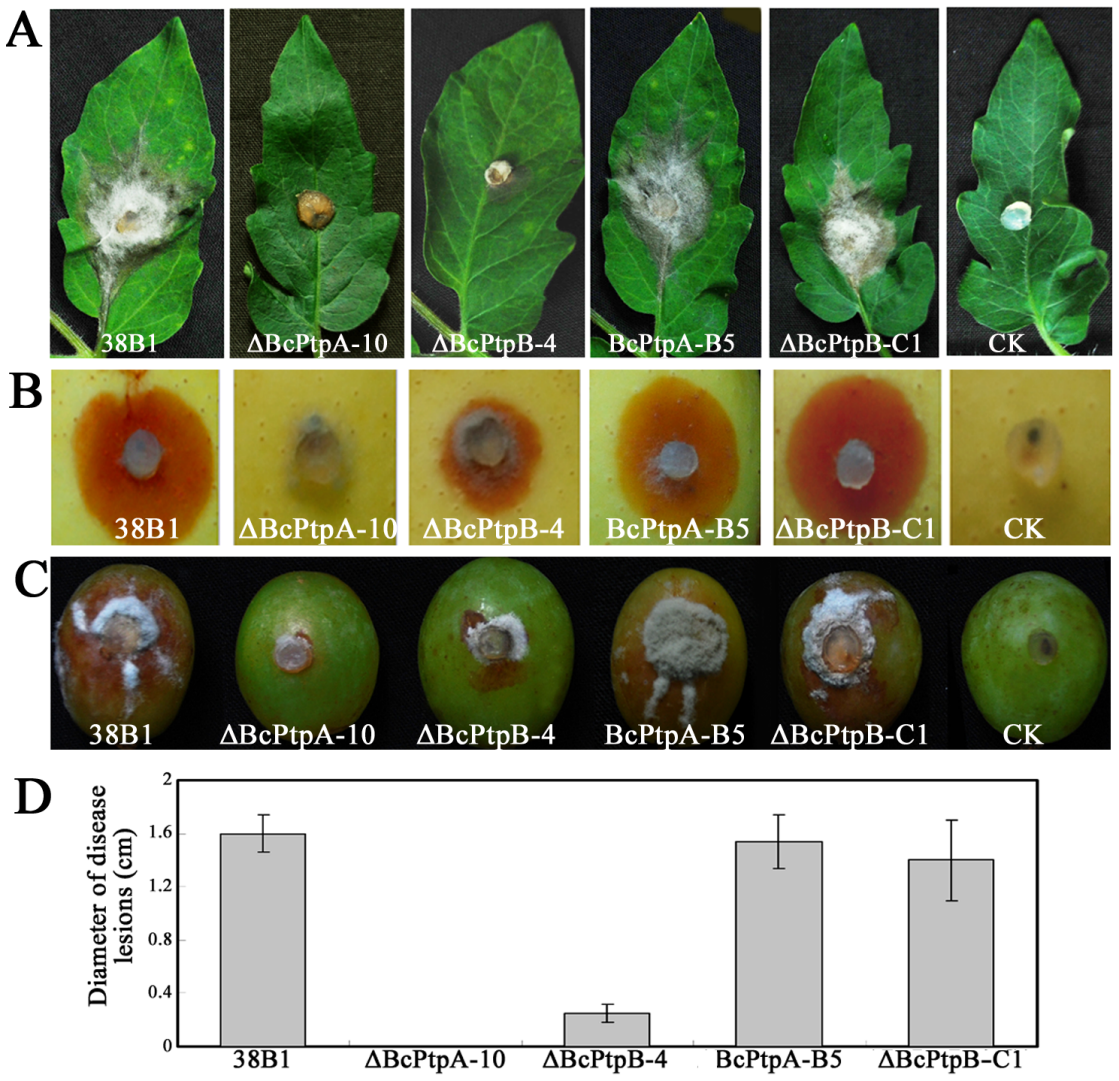
Pathogenicity assays on different plant tissues following inoculation with 38B1, ΔBcPtpA-10, ΔBcPtpB-4, BcPtpA-5, ΔBcPtpB-C1. Disease symptoms on wounded tomato leaves, 60 hours after inoculation (h.a.i.) (A), wounded apple fruits, 72 h.a.i.(B), wounded grape fruits, 72 h.a.i.(C). Diameter of disease lesions on tomato leaves caused by each strain, 60 h.a.i. (D). Agar plug without *B. cinerea* mycelia was used as a negative control (CK). Bars denote standard errors of four replications.

**Figure 12:**
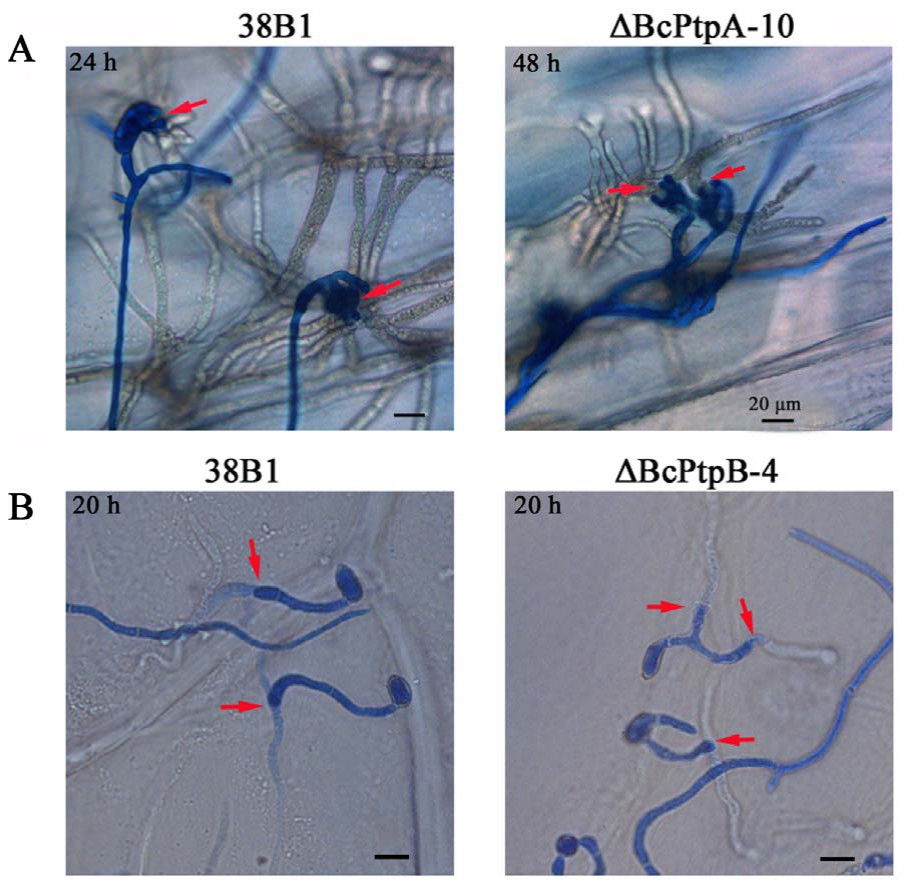
Onion penetration assay with 38B1, ΔBcPtpA-10 and ΔBcPtpB-4. Inoculation of chloroform treated onion epidermis with mycelium (**A**) or conidia **(B**). Penetration sites are indicated by red arrows. After incubation at 25°C for 20, 24 or 48 hours, onion epidermis was peeled and stained with cotton blue for microscopic examination.

At two days after inoculation, ΔBcPtpA-10 was unable to infect wounded tomato leaves at all, and ΔBcPtpB-4 caused significant smaller disease lesion than the wild-type 38B1 and the complemented strain ΔBcPtpB-C1 (Figures 11A, D). Similar results were observed on apple and grape fruits (Figures 11B, C). To analyze this pathogenicity defect of the mutants in details, onion epidermis penetration assay was performed. As shown in Figure 12A, mycelia of ΔBcPtpA-10 took 48 h to penetrate killed onion epidermis while the wild-type strain 38B1 could penetrate onion epidermis within 24 h after inoculation. Similar to the wild-type, conidia of ΔBcPtpB-4 were able to penetrate killed onion epidermis within 20 h of incubation (Figure 12B).

### Complementation of yeast *PTP2*, *PTP3* and *PTC1* deletion mutants with *BcPTPA* and *BcPTPB*

**Figure 13:**
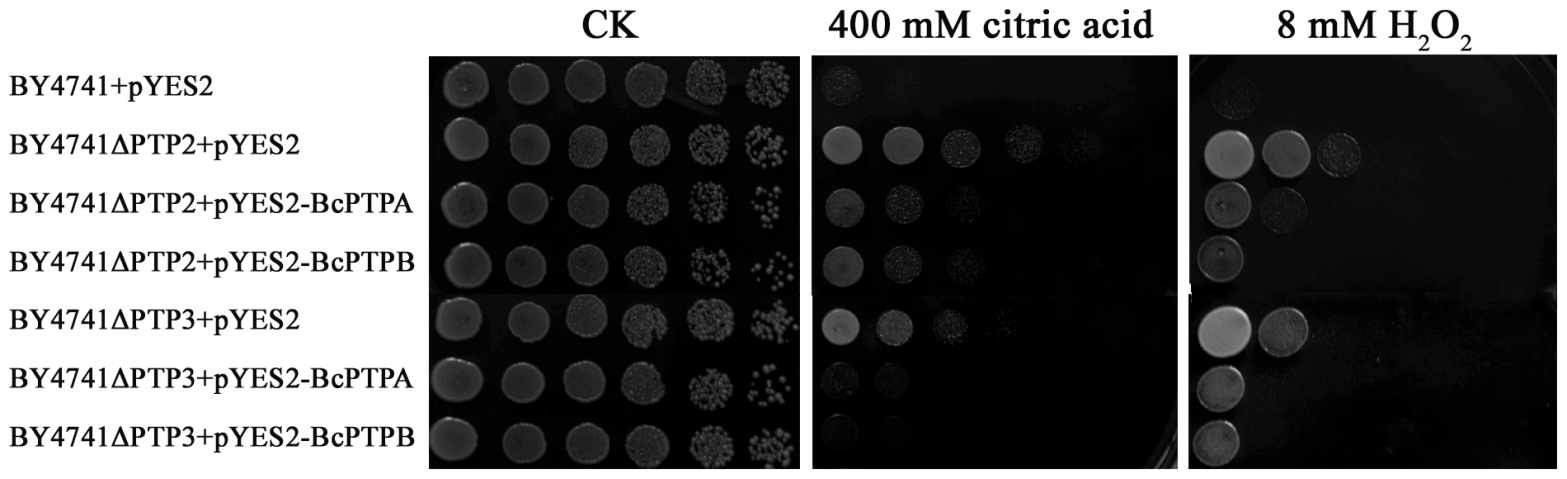
Complementation of *S. cerevisiae PTP2* and *PTP3* mutants with *BcPTPA* and *BcPTPB*. The *S. cerevisiae PTP2* and *PTP3* mutants were transformed with *BcPTPA* and *BcPTPB* cDNA to generate the strain BY4741ΔPTP2+pYES2-BcPTPA, BY4741ΔPTP2+pYES2-BcPTPB, BY4741ΔPTP3+pYES2-BcPTPA and BY4741ΔPTP3+pYES2-BcPTPB. The wild-type strain BY4741, BY4741ΔPTP2 and BY4741ΔPTP3 transformed with empty pYES2 vector were used as controls. Serial dilutions of cell suspension of each strain were spotted on YPRG plates under different stresses. After yeast cells were incubated at 30°C for four days, growth of each strain on each plate was examined.

In order to further determine functions of BcPtpA and BcPtpB, we tested whether *BcPTPA* and *BcPTPB* would complement the yeast *PTP2* and *PTP*3 mutants. Expression vector pYES2 containing the full-length *BcPTPA* or *BcPTPB* cDNA was transformed into the budding yeast *PTP2* and *PTP3* mutants BY4741ΔPTP2 and BY4741ΔPTP3. As a control, the mutant was also transformed with the empty pYES2 vector. As shown in Figure 13, the growth of BY4741ΔPTP2 and BY4741ΔPTP3 was significantly increased on YPRG medium amended with 400 mM citric acid and 8 mM H_2_O_2_. These phenotypes were restored by genetic complementation of yeast BY4741ΔPTP2 and BY4741ΔPTP3 mutants with *B. cinerea BcPTPA* and *BcPTPB* (Figure 13).

**Figure 14:**
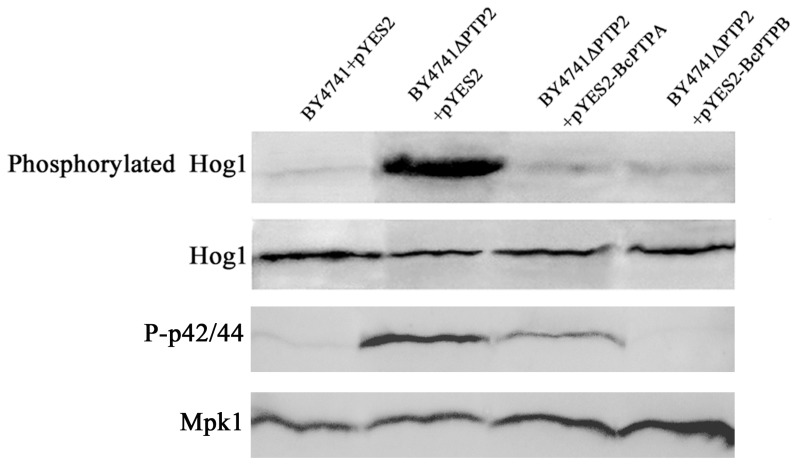
Comparison in phosphorylation of Hog1 and Mpk1 in the *S. cerevisiae* strains BY4741+ pYES2, BY4741ΔPTP2+ pYES2, BY4741ΔPTP2+pYES2-BcPTPA and BY4741ΔPTP2+pYES2-BcPTPB. Hog1 and phosphorylated Hog1 proteins were detected using the yeast anti-Hog1p (C-terminal anti-Hog1) and phosphorylated p38 (Thr180/Tyr182) antibodies, respectively.

In *S. cerevisiae*, both Ptp2 and Ptp3 inactivate Hog1 and Mpk1 although Ptp2 is a more effective negative regulator than Ptp3 [6], [7]. To further confirm the functions of *BcPTPA* and *BcPTPB* in *S. cerevisiae*, we examined phosphorylation of Hog1 and Mpk1 in BY4741+pYES2, BY4741ΔPTP2+pYES2, BY4741ΔPTP2+pYES2-BcPTPA and BY4741ΔPTP2+pYES2-BcPTPB. As shown in Figure 14, the basal phosphorylation level of Hog1 and Mpk1 in BY4741ΔPTP2+pYES2 was much higher than that in the wild-type strain and all the complemented mutants, indicating that both BcPtpA and BcPtpB could inactivate Hog1 and Mpk1 in *S. cerevisiae*. Additionally, BcPtpB is a more effective negative regulator of Mpk1 than BcPtpA (Figure 14).

**Figure 15:**
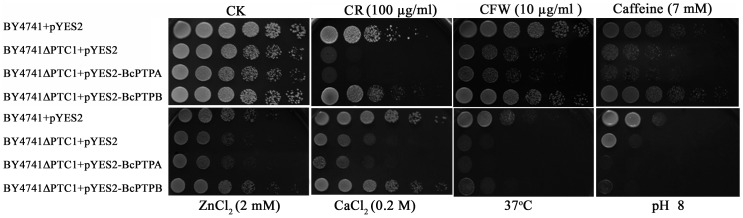
Complementation of *S. cerevisiae PTC1* mutant with *BcPTPA* and *BcPTPB*. The *S. cerevisiae PTC1* mutant was transformed with *BcPTPA* and *BcPTPB* cDNA to generate the strain BY4741ΔPTC1+pYES2-BcPTPA and BY4741ΔPTC1+pYES2-BcPTPB, respectively. The wild-type strain BY4741 and *PTC1* mutant BY4741ΔPTC1 transformed with empty pYES2 vector were used as controls. Serial dilutions of cell suspension of each strain were spotted on YPRG plates under different stresses. After yeast cells were incubated at 30°C or 37°C (as indicated) for four days, growth of each strain on each plate was examined.

In *S. cerevisiae*, Ptc1 is also a major negative regulator of HOG pathway, and *PTC1* deletion mutant showed significant phenotypic changes under various stress conditions [24], [25]. In order to further determine functions of BcPtpA and BcPtpB, we also tested whether *BcPTPA* and *BcPTPB* would complement the yeast *PTC1* mutants. As shown in Figure 15, the growth of BY4741ΔPTC1 was significantly hindered on YPRG medium amended with 100 µg/ml Congo red, 10 µg/ml calcofluor white (CFW), 0.5 M NaCl, 2 mM ZnCl_2_, or 0.2 M CaCl_2_. The growth defects were partially restored by genetic complementation of the budding yeast BY4741ΔPTC1 mutant with *B. cinerea BcPTPB* but not with *BcPTPA* (Figure 15). Additionally, the growth of BY4741ΔPTC1 was obstructed at high pH (8.0) or at 37°C, but this growth defect was not restored by genetic complementation of either *BcPTPA* or *BcPTPB*.

## Discussion

In *S. cerevisiae*, two protein tyrosine phosphatases, Ptp2 and Ptp3 play an important role in inaction of Hog1 in the HOG pathway [8]. In order to establish the role of BcPtpA and BcPtpB in the HOG pathway, in this study, we analyzed the phosphorylation profiles of the Hog1-like MAP kinase BcSak1 in *BcPTPA* and *BcPTPB* deletion mutants. Consistent with previous findings [26], [27], Western-blot analyses showed that BcSak1 were only weakly phosphorylated under standard conditions, and osmotic and oxidative stress treatments led to high levels of BcSak1 phosphorylation in the wild-type strain. However, the increased phosphorylation of BcSak1 were not observed in both mutants under osmotic and oxidative stresses, indicating that BcPtpA and BcPtpB do not acts as the phosphatases of BcSak1 in *B. cinerea,* which is opposite to that in *S. cerevisiae*. In budding yeast, phosphorylation levels of Hog1 were increased dramatically in *PTP2* or *PTP3* deletion mutants [8]. In addition, the yeast Hog1 physically interacts with Ptp2. There are two adjacent Pbs2-binding sites in Hog1, namely, the common docking (CD) domain and Pbs2-binding domain 2 (PBD-2). The CD and the PBD-2 docking sites play critical roles in both the activation and inactivation of Hog1 [28]. But in this study, we did not observe such interaction between BcSak1 and BcPtpA or BcPtpB in the yeast two-hybrid assays (Figure S2). These results indicate that the functions of BcPtpA and BcPtpB in the *B. cinerea* HOG pathway are different from those of their orthologs in *S. cerevisiae*.

A previous study showed that in the wild-type strain of *B. cinerea*, strong phosphorylation of BcSak1 was observed in response to osmotic stress (1 M NaCl), oxidative stress (10 mM H_2_O_2_) and fungicide treatments (25 µg/ml iprodione and 1 µg/ml fludioxonil), but not under standard conditions. However, in the two-component histidine kinase gene (*BOs-1*) deletion mutant, BcSak1 was highly phosphorylated regardless of the conditions tested [27], indicating Bos-1 is a negative regulator of BcSak1. Although *S. cerevisiae* contain is a histidine kinase, Sln1, in contrast to Bos-1, Sln1 has no N-terminal amino acid repeat domain, but contains two transmembrane regions [29], [30]. Interestingly, the antifungal activity of the fungicides iprodione and fludioxonil, which are very effective against filamentous fungi including *B. cinerea* and *Pyricularia oryzae*, is dependent on the presence of the two-component histidine kinase (os-1) in the HOG pathway [19]. However, these fungicides have no fungicidal effect on *S. cerevisiae* because the budding yeast doesn’t contain an os-1-like kinase. Surprisingly, expression of *OS-1* from *P. oryzae* can confer the sensitivity of *S. cerevisiae* to these fungicides [31], [32]. These results indicate that *S. cerevisiae* and filamentous fungi are significantly different in the component of histidine kinase in their HOG pathways.

In *B. cinerea*, Bos-1 is a negative regulator of BcSak1 [27]. In addition, Bos-1 is also involved in regulation of certain phenotypes in a BcSak1-indepent manner, such as tolerance to neutral hyperosmolarity, and to iprodione and fludioxonil, suggesting that other Bos1-dependent downstream partners could be responsible for these cellular functions [25], [33]. A recent study further showed that Bos-1 is also associated with the cell wall integrity in *B. cinerea* since *BOs-1* deletion mutant exhibited decreased sensitivity to the cell wall digesting enzymes, Glucanex. Moreover, in *BOs-1* mutant, the phosphorylation level of BcBmp3 (the ortholog of Slt2, which is a key MAP kinase in cell wall integrity signal pathway in *S. cerevisiae*) was higher than that in the wild-type strain [20]. In this study, we found that *BcPTPA* and *BcPTPB* deletion mutants revealed increased sensitivity to the Glucanex enzymes. Furthermore, the deletion of *BcPTPA* or *BcPTPB* led to undetectable levels of phosphorylated BcBmp3 in response to Congo red treatment. These observations indicate that BcPtpA and BcPtpB may be the negative regulators of Bos-1 in *B. cinerea.*

In this study, we found that BcPtpA and BcPtpB share several functions: 1) they both act as positive regulators of BcSak1 and BcBmp3 under stress conditions; 2) deletion of *BcPTPA* or *BcPTPB* results in increased pigmentation, and sensitivity to osmotic, oxidative and cell wall damage stresses, and leads to the defect of sclerotial formation. However, BcPtpA and BcPtpB have different roles in regulating of conidiation. The deletion of *BcPTPA*, but not *BcPTPB* gene, compromised the ability of *B. cinerea* conidiation on solid medium or plant tissue. Many previous studies have shown that conidiation of *B. cinerea* can be regulated by multiple signaling pathways including the VeA regulatory system [34], Ca^2+^/calcineurin-dependent signaling pathway [35], cAMP-dependent signaling pathway [36], and HOG signaling pathway [20], [26], [27]. Thus, BcPtpA and BcPtpB may target their unidentified specific downstream partners, which are involved in regulating of conidiation in *B. cinerea*. This deduction is further supported by the finding that *BcPTPB*, but not *BcPTPA*, can partially restore the growth defects of *S. cerevisiae PTC1* deletion mutant. However, additional experiments are necessary to identify the specific substrates of BcPtpA and BcPtpB in *B. cinerea*.

In this study, *BcPTPA* and *BcPTPB* deletion mutants exhibited dramatically decreased virulence, which may result from multiple defects of the mutants. First, the mutants grew slower than the parental strain. Second, these mutants showed increased sensitivity to H_2_O_2_ that could be produced by host plants in response to fungal infection [37]. Tolerance to oxidative burst, characterized by a strong accumulation of reactive oxygen species has been considered to be an important element of *B. cinerea* to infect plant tissue [38]–[40]. Third, the deletion of *BcPTPA* and *BcPTPB* leads to increased sensitivity of *B. cinerea* to cell wall-damaging agents. Previous studies have showed that cell wall integrity is required for *B. cinerea* virulence because weaken cell wall leads to reduced virulence [41], [42]. In addition, osmo-adaptation may be potential involved in *B. cinerea* infection process [33], [43]. Increased sensitivity of the mutants to osmotic stress may also compromise the ability of *B. cinerea* to host plant.

## Materials and Methods

### Fungal strain and culture condition

*B. cinerea* strain 38B1 isolated from grape was used as a recipient strain for the transformation experiments. This strain was deposited in the China Microbiological Culture Collection Center, under accession number CGMCC No. 4006.

*B. cinerea* was grown on potato dextrose agar (PDA) (200 g potato, 20 g glucose, 20 g agar, and 1 L water), minimal medium (MM) (10 mM K_2_HPO_4_, 10 mM KH_2_PO_4_, 4 mM (NH_4_)_2_SO_4_, 2.5 mM NaCl, 2 mM MgSO_4_, 0.45 mM CaCl_2_, 9 µM FeSO_4_, 10 mM glucose, and 1 L water, pH 6.9) and on sterilized cucumber fragments for mycelial growth and conidiation tests, respectively.

Mycelial growth tests under different conditions were performed on PDA and MM plates with the following supplements: the osmotic agents NaCl and D-sorbitol; oxidative stress generators H_2_O_2_ and paraquat; the antifungal compounds iprodione and fludioxonil (96.5% a.i., Heyi Agricultural Chemical Co. Ltd., Zhejiang, China); and cell wall damaging agents Caffeine and Congo red at concentrations as indicated in the figure legends. Each plate was inoculated with a 5-mm diameter mycelial plug taken from the edge of a 3-day-old colony grown on PDA. After the plates were incubated at 25°C for 2 days, colony diameter in each plate was measured with the original mycelial plug diameter subtracted from each measurement. The percentage of mycelial radial growth inhibition (RGI) was calculated using the formula RGI% = ((C–N)/(C–5))*100, where, C is colony diameter of the control without any treatment, and N is that of a treatment. The experiments were repeated three times.

### Sequence analysis of *BcPTPA* and *BcPTPB*

*BcPTPA* (XP_001553725.1) and *BcPTPB* (XP_001552511.1) was originally identified by homology search of the *B. cinerea* genome sequence (http://www.broad.mit.edu/annotation/genome/botrytis_cinerea/Home.html) using BLASTP algorithm with the Ptp2 and Ptp3 protein from *S. cerevisiae* [8] as queries. To verify the existence and size of the introns, RNA was extracted from mycelia of the wild-type strain 38B1 with a TaKaRa RNAiso Reagent (TaKaRa Biotech. Co., Dalian, China) and used for reverse transcription with a RevertAid H Minus First Strand cDNA Synthesis kit (Fermentas Life Sciences, Burlington, Canada) according to the manufacturer's instructions. Reverse transcription PCR was performed with the primer pair BcPtpA-F and BcPtpA-R, BcPtpB-F and BcPtpB-R, respectively (Table S1). The resultant PCR product was purified, cloned and sequenced.

### Construction of *BcPTPA* and *BcPTPB* deletion and complemented mutants

*BcPTPA* deletion vector pCA-BcPtpA-Del was constructed by inserting two flanking sequences of *BcPTPA* into two sides of the *HPH* (hygromycin resistance) gene in the pBS-HPH1 vector [44]. A 928-bp upstream flanking sequence fragment of *BcPTPA* amplified from 38B1 genomic DNA using the primer pair BcPtpA-up-F and BcPtpA-up-R was inserted into *Xho* I-*Sal* I sites of the pBS-HPH1 vector to generate the plasmid pBS-BcPtpA-up. Subsequently, a 937-bp downstream flanking sequence fragment of *BcPTPA* amplified from 38B1 genomic DNA using the primer pair BcPtpA-down-F and BcPtpA-down-R was inserted into *Hind* III-*BamH* I sites of the pBS- BcPtpA-up vector to generate the plasmid pBS- BcPtpA-UD. Finally, the 3,365-bp fragment containing BcPtpA-upstream-HPH- BcPtpA-downstream cassette was obtained by digestion of the plasmid pBS-PtpA-UD with *Xho* I and *BamH* I, and ligated into the *Xho* I-*BamH* I sites of pCAMBIA 1300 (CAMBIA, Canberra, Australia). The resultant *BcPTPA* gene deletion vector pCA- BcPtpA-Del (Figure S1A) was transformed into the *Agrobacterium tumefaciens* strain C58C1. *BcPTPB* deletion vector pCA-BcPtpB-Del was constructed using the same strategy.

The *A. tumefaciens*-mediated fungal transformation was performed as described previously [45]. Briefly, *A. tumefaciens* strain C58C1 containing an appropriate binary vector, was grown at 28°C for 2 days in minimal medium (MM) supplemented with kanamycin (100 µg/ml). *A. tumefaciens* cells were diluted to an optical density with OD_600_ = 0.15 in induction medium (IM) containing 200 µM acetosyringone (AS). The cells were grown for additional 6 h before mixing them with an equal volume of fresh *B. cinerea* conidial suspension (1×10^6^ conidia per ml). A 200 µl aliquot of the mix was sprayed on each piece of nylon membrane (3×3 cm) (Millipore Co., Bedford, MA, USA), and plated on IM amended with 200 µM AS. After incubation at 20°C for 2 days in the dark, the membrane was cut into small pieces (3×0.1 cm), and transferred upside-down on PDA plates supplemented with hygromycin B (100 µg/ml) as a selection agent for transformants and cefotaxime (200 µM) to kill the *A. tumefaciens* cells. After 5 to 7 days of incubation, hygromycin resistant colonies appeared and individual transformants were transferred onto PDA plates amended with hygromycin B at 100 µg/ml.

The complementation plasmid pCA-BcPtpB-C was constructed on the backbone of pCAMBIA1300. First, the chlorimuron-ethy1 resistance gene (*SUR*) was amplified from plasmid PCB1532 [46] with the primer pair SUR-F and SUR-R, and cloned into the *Sal* I site of pCAMBIA1300 to create plasmid pCA-SUR. Then, the complete *BcPTPB* gene including 2,981-bp upstream and 254-bp terminator region was amplified from genomic DNA of the wild-type strain with the primer pair BcPtpB-com-F and BcPtpB-com-R, and cloned into the *Pst* I and *Sac* II site of pCA-Sur to generate a complementation plasmid pCA-BcPtpB-C. Before the plasmid pCA-BcPtpB-C was transformed into *A. tumefaciens* strain C58C1, *BcPTPB* was sequenced to ensure flawlessness of the sequence. Transformation of ΔBcPtpB-4 with pCA-BcPtpB-C was conducted as described above except that hlorimuron-ethy1 was used as a selection agent. For complementation of the mutant ΔBcPtpA-10, because the publicly available *B. cinerea* genome sequence is incomplete, we were not successful in amplifying the promoter region of *BcPTPA* using the thermal asymmetric interlaced PCR (TAIL-PCR) method [47]. Thus, an ectopic mutant ΔBcPtpA-5 was selected as an alternative approach.

### Expression analysis of a melanin biosynthesis related gene *THR1*

Expression levels of *THR1* gene in each strain were measured by real-time PCR assay. Briefly, each strain was grown in potato dextrose broth at 25°C for 3 days in a shaker. Mycelia of each strain were harvested and ground in liquid nitrogen. RNA extraction and reverse transcription was performed using the protocol described above. The real-time PCR amplifications were conducted in a DNA Engine Opticons 4 System (MJ Research, Inc., Waltham, MA, USA) using TAKARA SYBR Premix Ex Taq (TAKARA Bio Inc., Dalian, China). There were two replicates for each sample. For each sample, PCR amplifications with primer pair β-tubulin-F and β-tubulin-R for the quantification of expression of β-tubulin gene were performed as a reference. The experiment was repeated three times. Gene expression levels were calculated using the 2^−ΔΔCt^ method [48].

### Intracellular glycerol accumulation

Glycerol accumulation in mycelia of each strain was measured using a previous published method [49]. Briefly, each strain was grown in potato dextrose broth for 2 days at 25°C in a shaker. After addition of 0.5 M NaCl and further incubation for 2 h, mycelia of each strain were harvested and ground in liquid nitrogen. The glycerol concentration was measured as described previously [26], [49].

### Western-blot analysis

Each strain was grown in potato dextrose broth at 25°C for 2 days in a rotary shaker. After the cultures were treated with 0.5 M NaCl, 24 mM H_2_O_2_ or 0.3 mg/ml Congo red for 2 h, mycelia of each strain were harvested and ground in liquid nitrogen. The extraction of protein and Western blot was performed as described [20], [26]. For detection of BcSak1, an anti-Hog1 antibody (C-terminal anti-Hog1) from Santa Cruz Biotechnology (CA, USA) was used. Phosphorylation of BcSak1 in *B. cinerea* was examined by using an antibody against dually phosphorylated p38 (Thr180/Tyr182) (Cell Signaling Technology, Beverly, MA, USA). Phospho-p44/42 MAP kinase antibody (Cell Signaling Technology, Beverly, MA, USA) was used to detect the phosphorylated (Thr/Tyr) of the *B. cinerea* MAP kinases BcBmp3 [50]. The yeast anti-Mpk1 (yN-19) from Santa Cruz Biotechnology (CA, USA) was used for detection of BcBmp3.

### Pathogenicity assays

Leaves of three-week-old rapeseed and tomato plants, and grape and apple fruits were inoculated with 5 mm diameter plugs of 4-day-old cultures. Before inoculation, leaves and fruits were wounded with a sterilized needle tip to facilitate penetration of the fungus into plant tissue. Inoculated tissues were incubated at 25°C with 16 h of daylight for up to four days. Diameter of disease lesions was recorded for each leaf at two days after inoculation. The experiment was repeated four times.

Infection-related morphogenesis was observed on onion epidermis as previously described [33]. Conidial suspensions (5×10^3^ conidia ml^−1^) or mycelia plugs were deposited onto the hydrophobic side of the epidermis. After 20 h or 48 h of incubation in a humid environment at 25°C, the epidermis was stained with aniline blue before microscopic evaluation [51]. Fungal mycelia were observed under light transmission microscopy.

### Complementation of yeast mutants with *BcPTPA* and *BcPTPB*

The yeast strain BY4741 (wild type), *PTC1* deletion mutant BY4741ΔPTC1, *PTP2* deletion mutant BY4741ΔPTP2, and *PTP3* deletion mutant BY4741ΔPTP3 were ordered from EUROSCARF (http://web.uni-frankfurt.de/fb15/mikro/euroscarf/). The full-length *BcPTPA* cDNA was amplified using the primer pair YES2-PtpA-F and YES2-PtpA-R. The PCR product was digested with *BamH* I and *Kpn* I, cloned into the pYES2 vector (Invitrogen), and transformed into the yeast mutant BY4741ΔPTC1, BY4741ΔPTP2, and BY4741ΔPTP3. Yeast transformants were selected on synthetic medium lacking uracil (Clontech). Additionally, the wild-type strain BY4741, BY4741ΔPTC1, BY4741ΔPTP2 and BY4741ΔPTP3 mutants transformed with empty pYES2 vector were used as controls. The pYES2-BcPTPB was constructed using the same strategy as the pYES2-BcPTPA was constructed. For the complementation assays, the yeast transformants were grown on YPRG medium (1% yeast extract, 2% peptone, 1% galactose, 1% raffinose, 2% agar) supplied with various stress agents including citric acid, H_2_O_2_, Congo red (CR), calcofluor white (CFW), NaCl, ZnCl_2_, CaCl_2_, and pH 8 at concentrations indicated in figure legends. The experiments were repeated three times.

### Yeast two-hybrid analysis

To construct plasmids for yeast two hybrid screen analysis, the coding sequence of the full length *BcPTPA*, *BcPTPB*, *BcSAK1* and *BcBMP3* was amplified from cDNA of the wild-type strain. The gene fragments were inserted into the *Nde* I-*BamH* I sites of the yeast GAL4 binding domain vector pGBKT7 and GAL4 activation domain vector pGADT7 (Clontech, Mountain View, CA, USA). The yeast two hybrid plasmids pGADT7-BcPtpA+pGBKT7-BcSak1, pGADT7-BcPtpB+pGBKT7-BcSak1, pGADT7-BcPtpA+pGBKT7-BcBmp3, pGADT7-BcPtpB+pGBKT7-BcBmp3, were co-transformed into the *S. cerevisiae* reporter strain AH109 according to LiAc/SS-DNA/PEG transformation procedure [52]. In-frame fusion was confirmed by sequencing. The pair of plasmid pGBKT7-53 (encoding a fusion of the DNA binding domain with murine p53 protein) and pGADT7 was served as a positive control. The pairs of plasmids pGBKT7-Lam (encoding a fusion of the DNA binding domain with human lamin C) and pGADT7, was used as a negative control. Transformants were grown at 30°C for 72 h on synthetic medium lacking leucine and tryptophane, and then transferred to the medium lacking histidine, leucine, and tryptophane but containing 5 mM 3-aminotriazole (3-AT) to identify binding activity. Each experiment was conducted in triplicate.

## Supporting Information

Figure S1 **Generation and identification of** ***BcPTPA*** **and** ***BcPTPB*** **deletion mutants.** (**A**) Gene replacement strategy for *BcPTPA.* Primer (codes 1-8) binding sites are indicated by arrows (see Table S1 for the primer sequences). (**B**) Gene replacement strategy for *BcPTPB*. Primer (codes 9-16) binding sites are indicated by arrows (see Table S1 for the primer sequences). (**C**) Southern blot hybridization analysis of transformants using the upstream of *BcPTPA* as a probe. Genomic DNA preparations of 38B1, ΔBcPtpA-2, ΔBcPtpA-10, and BcPtpA-5 were digested with *Nde* I. (**D**) Southern blot hybridization analysis of transformants using hygromycin resistance gene (*HPH*) as a probe. Genomic DNA preparations of 38B1, ΔBcPtpA-2 and ΔBcPtpA-10 were digested with *Sac* I. (**E**) Southern blot hybridization analysis of transformants using the upstream of *BcPTPB* as a probe. Genomic DNA preparations of 38B1, ΔBcPtpB-4 and ΔBcPtpB-C1 were digested with *Sca* I.
(TIF)

Figure S2 **Yeast two-hybrid analysis of the interaction between BcPtpA, BcPtpB and BcSak1, BcBmp3.** The pair of plasmids pGBKT7-53 and pGADT7 served as a positive control. The pair of plasmids pGBKT7-Lam and pGADT7 was used as negative control. Growth of each yeast strain was assayed on medium containing 5 mM 3-aminotriazole [3-AT], but lacking histidine, leucine and tryptophane. Columns in each panel represent serial decimal dilutions.
(TIF)

Table S1 PCR primers used in this study.
(DOC)
