# Correction: Involvement of Protein Tyrosine Phosphatases BcPtpA and BcPtpB in Regulation of Vegetative Development, Virulence and Multi-Stress Tolerance in *Botrytis cinerea*

**DOI:** 10.1371/journal.pone.0151720

**Published:** 2016-03-14

**Authors:** Qianqian Yang, Fangwei Yu, Yanni Yin, Zhonghua Ma

Some images in this PLOS ONE paper are duplicated in the following article published in the journal Environmental Microbiology:

Yang, Q., Jiang, J., Mayr, C., Hahn, M. and Ma, Z. (2013), Involvement of two type 2C protein phosphatases BcPtc1 and BcPtc3 in the regulation of multiple stress tolerance and virulence of *Botrytis cinerea*. Environ Microbiol, 15: 2696–2711. doi:10.1111/1462-2920.12126 [2]

Control plates shown in the Environmental Microbiology article Figure 2A and in the PLOS ONE article Fig 4A for 38B1 + 50ug are the same. Control plates shown in the Environmental Microbiology article Figure 7 Panel C and in the PLOS ONE Figure 11 Panel C for 38B1 and CK are the same. The authors would like to clarify that the mutants BcPtc1/3 (described in Environmental Microbiology) and the mutants BcPtpA/B (described in PLOS ONE) were generated from the same parental strain 38B1; since the mutants were characterised at the same times, the same control plates were used.

There is an error in Fig 4A of the PLOS ONE paper that arose due to a mistake in photographing the plates. The plates shown in the Environmental Microbiology article Figure 2A for ΔBcPtc1-C1–50μg tricyclazole and in the PLOS ONE article Fig 4A for ΔBcPtpA-5–50μg tricyclazole are the same. In order to address this error, the authors have reactivated the strains to repeat the experiments. Here we provide a revised Fig 4 using the newly generated plates.

**Fig 4:**
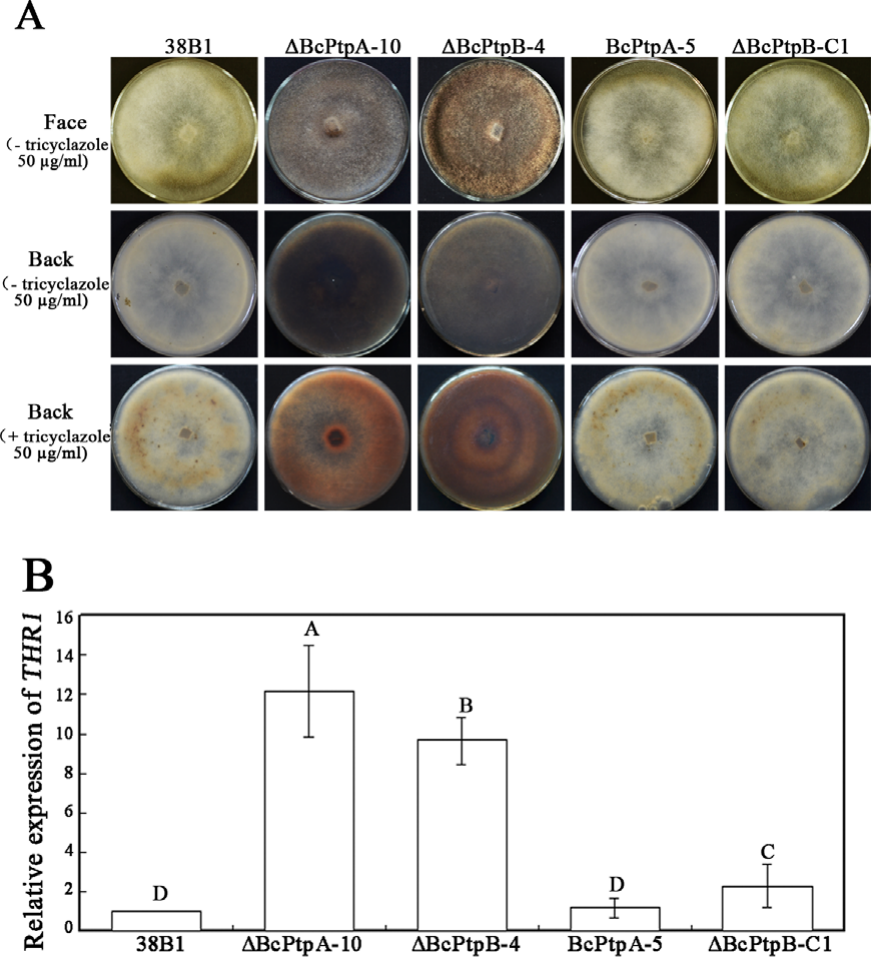
Involvement of *BcPTPA* and *BcPTPB* in the regulation of hyphal melanization. (A) Comparisons of mycelial melanization among the wild-type strain 38B1, ΔBcPtpA-10, ΔBcPtpB-4, BcPtpA-5 and ΔBcPtpB-C1 after 9 days of incubation in darkness on PDA plates amended with or without 50 μg/ml tricyclazole. (B) Relative expression level of *THR1* (1,3,8-trihydroxynaphthalene reductase gene), which is involved in melanin biosynthesis. Bars denote standard errors from three replications. Values on the bars followed by the same letter are not significantly different at *P* = 0.05.

In Figure 14 of the PLOS ONE paper, lanes from the same blot were spliced together, but the splicing was not clearly indicated. Lanes 2–4 of the original blot were removed as they contained data that was not included in the PLOS ONE paper. The original lanes in the blot can be seen in the photo uploaded as Supporting Information.

The raw, uncropped blot images were not available to publish as Supporting Information. In the absence of these original files, the authors have repeated the experiments for Figures 9, 10, and 14. Here we provide the raw, uncropped blots generated in these repeat experiments as Supporting Information.

## Supporting Information

S1 File Results from repeated experiments for Figures 9, 10, and 14. (ZIP)

S1 Photo Original blot for Figure 14. (JPG)
