# Supplementary figures and images for: Involvement of Protein Tyrosine Phosphatases BcPtpA and BcPtpB in Regulation of Vegetative Development, Virulence and Multi-Stress Tolerance in Botrytis cinerea
Source: PLoS One. 2013 Apr 9;8(4):e61307. doi: 10.1371/journal.pone.0061307 (PMC3621866; doi:10.1371/journal.pone.0061307)

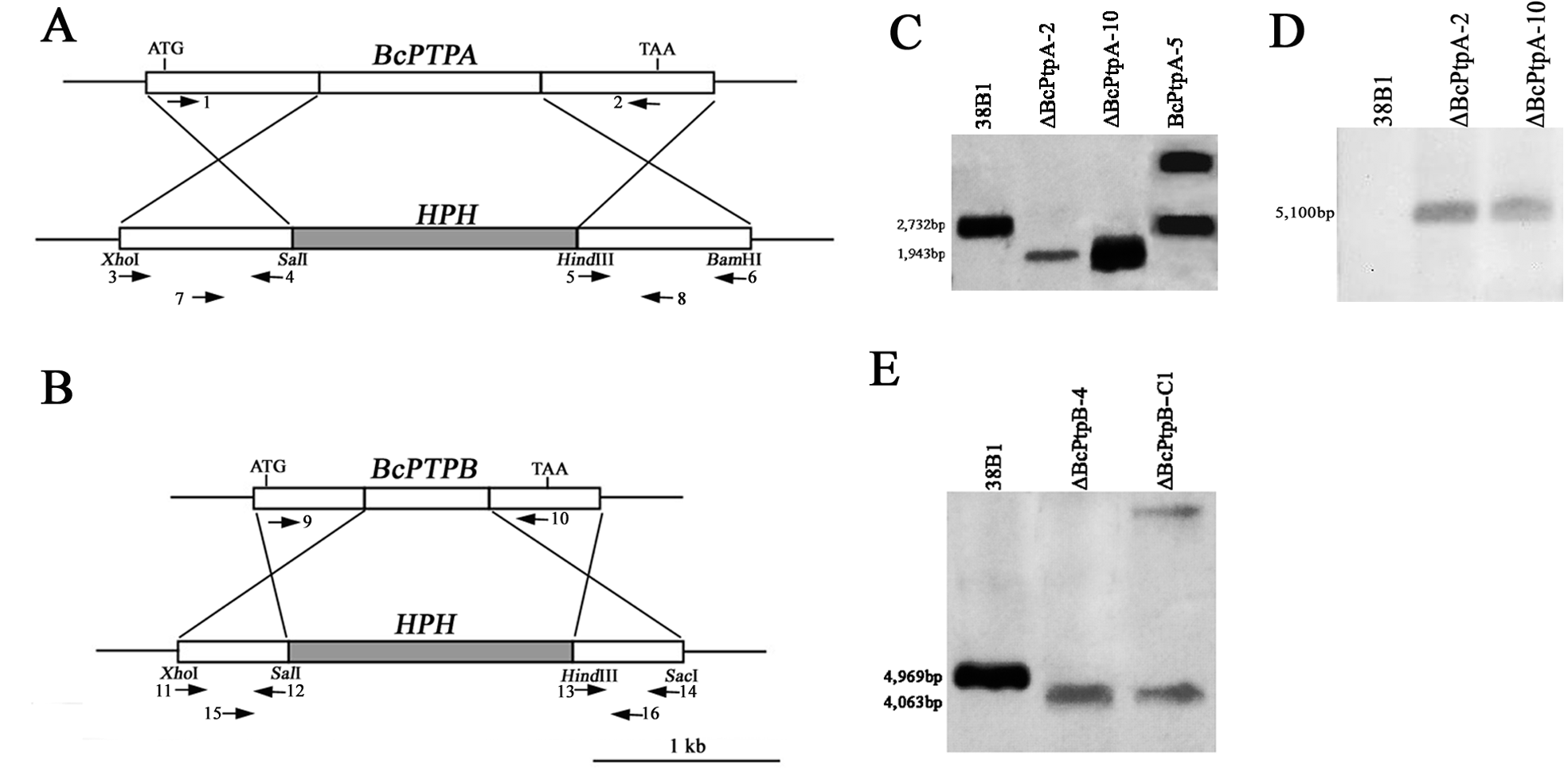

Supplement: Figure S1 — Generation and identification of BcPTPA and BcPTPB deletion mutants. (A) Gene replacement strategy for BcPTPA. Primer (codes 1-8) binding sites are indicated by arrows (see Table S1 for the primer sequences). (B) Gene replacement strategy for BcPTPB. Primer (codes 9-16) binding sites are indicated by arrows (see Table S1 for the primer sequences). (C) Southern blot hybridization analysis of transformants using the upstream of BcPTPA as a probe. Genomic DNA preparations of 38B1, ΔBcPtpA-2, ΔBcPtpA-10, and BcPtpA-5 were digested with Nde I. (D) Southern blot hybridization analysis of transformants using hygromycin resistance gene (HPH) as a probe. Genomic DNA preparations of 38B1, ΔBcPtpA-2 and ΔBcPtpA-10 were digested with Sac I. (E) Southern blot hybridization analysis of transformants using the upstream of BcPTPB as a probe. Genomic DNA preparations of 38B1, ΔBcPtpB-4 and ΔBcPtpB-C1 were digested with Sca I. (TIF) [file pone.0061307.s001.tif]

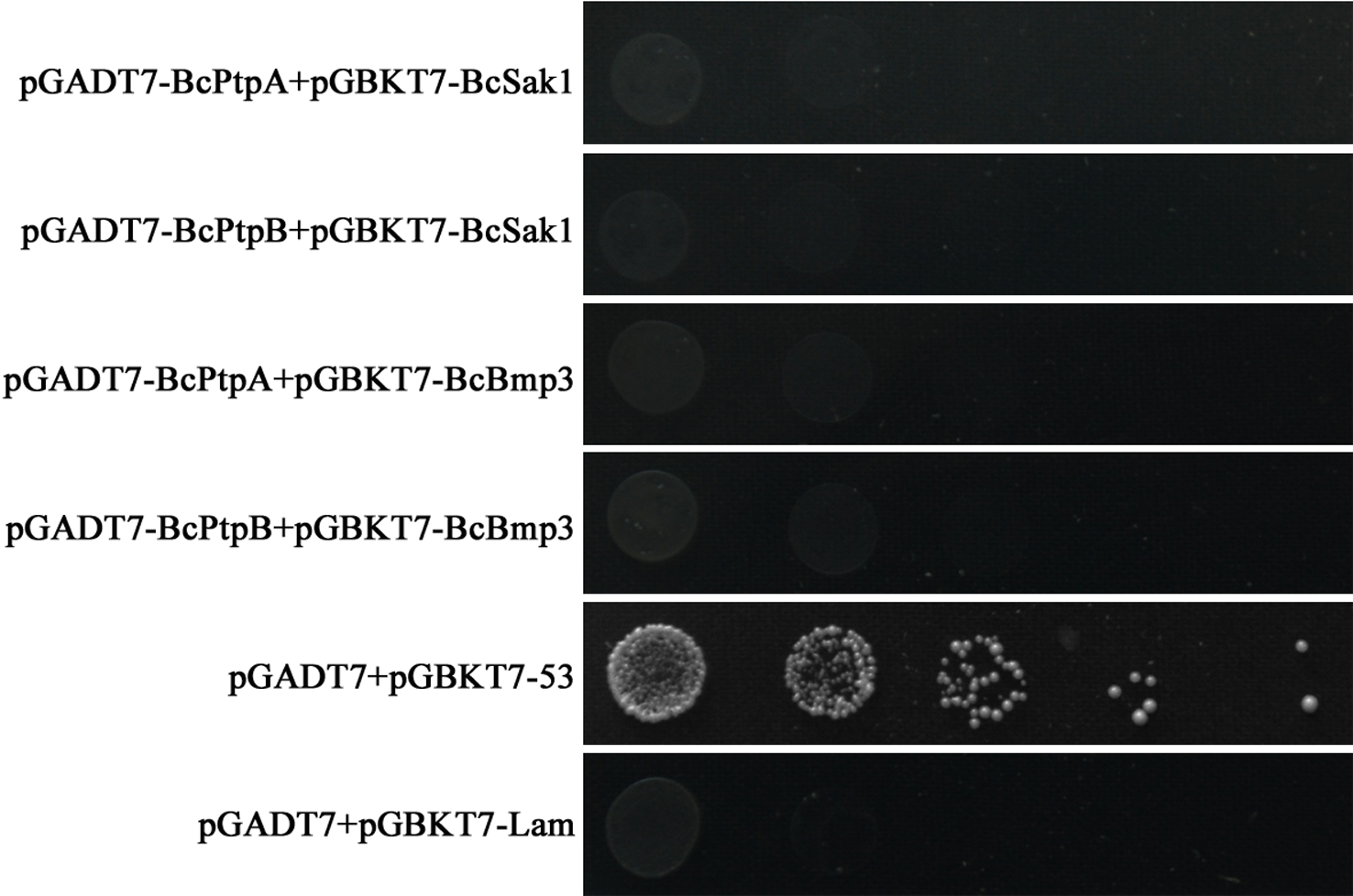

Supplement: Figure S2 — Yeast two-hybrid analysis of the interaction between BcPtpA, BcPtpB and BcSak1, BcBmp3. The pair of plasmids pGBKT7-53 and pGADT7 served as a positive control. The pair of plasmids pGBKT7-Lam and pGADT7 was used as negative control. Growth of each yeast strain was assayed on medium containing 5 mM 3-aminotriazole [3-AT], but lacking histidine, leucine and tryptophane. Columns in each panel represent serial decimal dilutions. (TIF) [file pone.0061307.s002.tif]
