# Supplementary material for: Involvement of Protein Tyrosine Phosphatases BcPtpA and BcPtpB in Regulation of Vegetative Development, Virulence and Multi-Stress Tolerance in Botrytis cinerea
Source: PLoS One. 2013 Apr 9;8(4):e61307. doi: 10.1371/journal.pone.0061307 (PMC3621866; doi:10.1371/journal.pone.0061307)
Supplement: Table S1 — PCR primers used in this study. (DOC) [file pone.0061307.s003.doc]

**Table S1.** PCR primers used in this study

| **Primercode** | **Primer** | **Sequence (5’-3’)** | **Relevant characteristics** |
| --- | --- | --- | --- |
| 1 | BcPtpA-F | ATATGAGTCCGGCAACAGGGA | PCR primers for amplification of full cDNA sequence of *BcPTPA* gene |
| 2 | BcPtpA-R | TTAGTATTGATACTCACTCAAAC |
|  |  |  |  |
| 3 | BcPtpA-up-F | ATctcgagAAAAAGGATAAAGGTTTTGCG | PCR primers to amplify *BcPTPA* upstream fragment for the construction of *BcPTPA* deletion mutants |
| 4 | BcPtpA-up-R | ATgtcgacCAGTTGCGGTAATCCTGGC |
|  |  |  |  |
| 5 | BcPtpA-down-F | ATaagcttATCCGAAAAGAAAGTCCACC | PCR primers to amplify *BcPTPA* downstream fragment for the construction of *BcPTPA* deletion mutants |
| 6 | BcPtpA-down-R | ATggatccTGAAGGAAGGGAAGGAAGGTA |
|  |  |  |  |
| 7 | BcPtpA-out-F | CCCTAGGCTTTCTTTACCTCA | PCR primers for identification of *BcPTPA* deletion transformants |
| 8 | BcPtpA- out-R | TAGTACTTGCGCGTTTTCGT |
|  |  |  |  |
| 9 | BcPtpB-F | ATGAACGCGATTAATAATAACGA | PCR primers for amplification of full cDNA sequence of *BcPTPB* gene |
| 10 | BcPtpB-R | TCACGCATGAGGAGATTTGA |
|  |  |  |  |
| 11 | BcPtpA-up-F | ATctcgagCAATCACAACCACAATCGCA | PCR primers to amplify *BcPTPB* upstream fragment for the construction of *BcPTPB* deletion mutants |
| 12 | BcPtpB-up-R | ATgtcgacCATTTCCCAAAACAATCCCC |
|  |  |  |  |
| 13 | BcPtpB-down-F | ATaagcttTCTGGATGGGTTATCGAAAGA | PCR primers to amplify *BcPTPB* downstream fragment for the construction of *BcPTPB* deletion mutants |
| 14 | BcPtpB-down-R | ATgagctcCAACCAAGCCAGCCTAATAA |
|  |  |  |  |
| 15 | BcPtpB-out-F | AAATGGAAGTGGAGAGGAGCA | PCR primers for identification of *BcPTPB* deletion transformants |
| 16 | BcPtpB-out-R | CGCAACTCTATGCACCATTAA |
|  |  |  |  |
| 17 | BcPtpB-Com-F | ATctgcagGCAAGGAGGGGTATTTATGCT | PCR primers to amplify full *BcPTPB* including 2,981-bp up and 254-down fragment for complement of the *BcPTPB* deletion mutant |
| 18 | BcPtpB-Com-R | ATccgcggCGCATGAGGAGATTTGACAC |
|  |  |  |  |
| 19 | YES2-BcPtpA-F | ATggtaccATATGAGTCCGGCAACAGGGA | PCR primers for amplification of full cDNA sequence of *BcPTPA* gene for construction of YES2- *BcPTPA* |
| 20 | YES2-BcPtpA-R | ATggatccTTAGTATTGATACTCACTCAAAC |
|  |  |  |  |
| 21 | YES2-BcPtpB-F | ATgaattcATGAACGCGATTAATAATAACGA | PCR primers for amplification of full cDNA sequence of *BcPTPB* gene for construction of YES2- *BcPTPB* |
| 22 | YES2-BcPtpB-R | ATtctagaTCACGCATGAGGAGATTTGA |
|  |  |  |  |
| 23 | SUR-F | ACGTGCCAACGCCACAGT | PCR primers for amplification of chlorimuron-ethy resistance gene (*SUR*) |
| 24 | SUR-R | ACGTGAGAGCATGCAATTCC |
|  |  |  |  |
| 25 | β-tubulin-F | ACCGTTCCAGAGTTGACTCAA | PCR primers to amplify *β-tubulin* downstream fragment for the expression levels analysis |
| 26 | β-tubulin-R | GCAAGAAAGCCTTTCTTCTGA |
|  |  |  |  |
| 27 | THR1-ex-F | TCTGGTTCTAAGGGTGCCATT | PCR primers to amplify *THR1* downstream fragment for the expression levels analysis |
| 28 | THR1-ex-R | CCTTTCCGTTAACCCATTCA |

The respective exogenous enzyme sites are lowercase in the sequence.
