# Supplementary figures and images for: Correction: Involvement of Protein Tyrosine Phosphatases BcPtpA and BcPtpB in Regulation of Vegetative Development, Virulence and Multi-Stress Tolerance in Botrytis cinerea
Source: PLoS One. 2016 Mar 14;11(3):e0151720. doi: 10.1371/journal.pone.0151720 (PMC4790925; doi:10.1371/journal.pone.0151720)

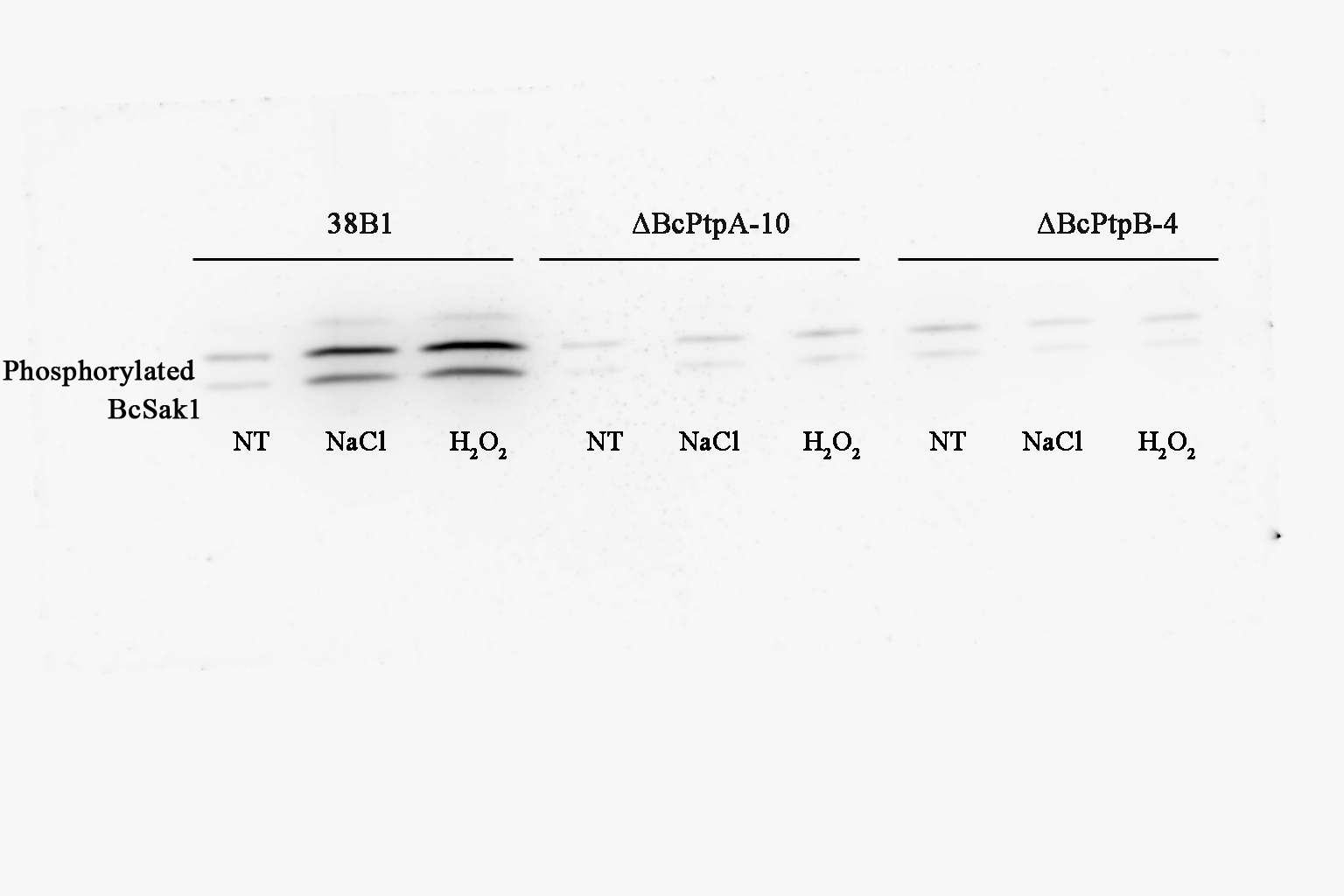

Supplement: S1 File — (ZIP) [file pone.0151720.s001.zip › Fig 9 Upper Panel.tif]

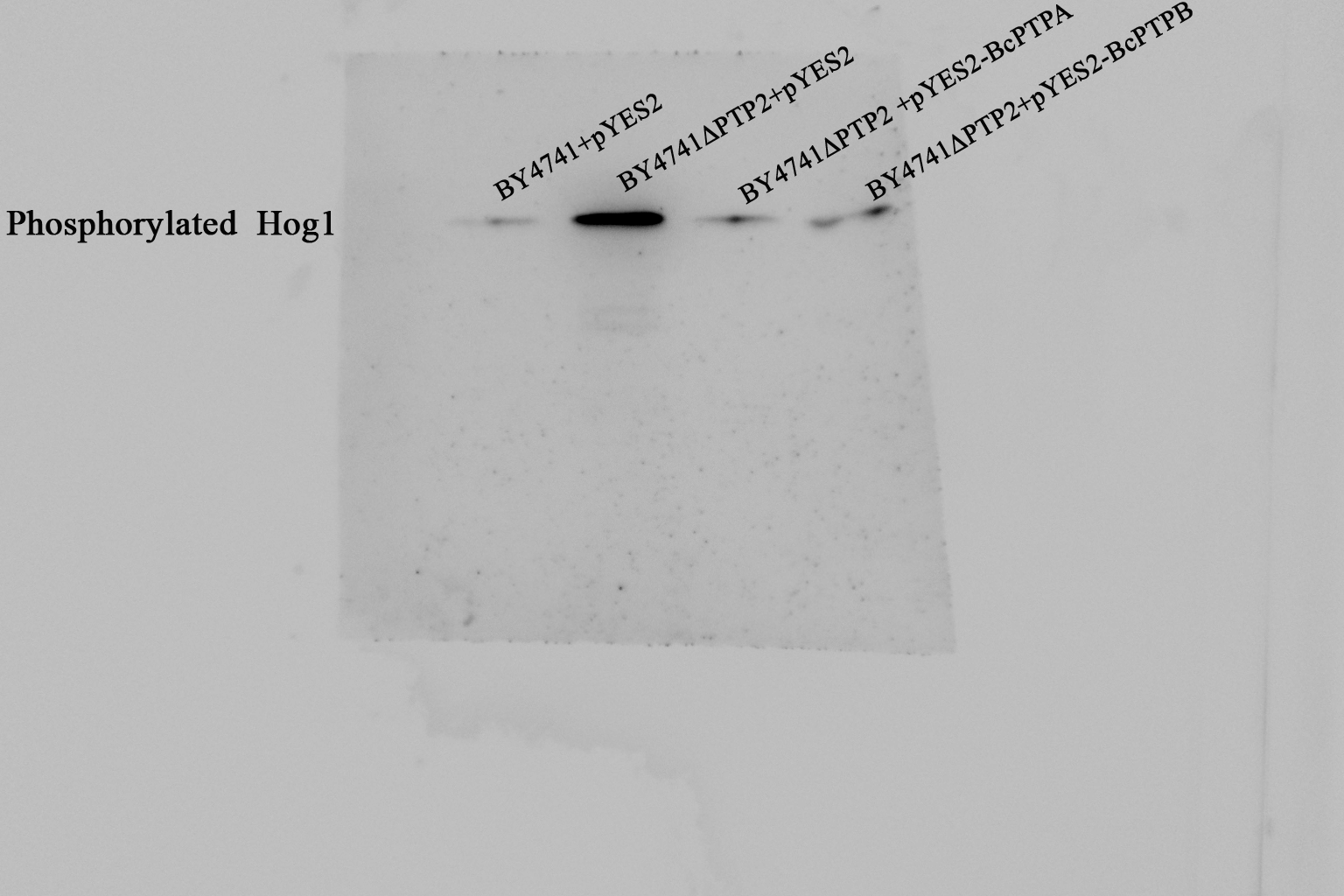

Supplement: S1 File — (ZIP) [file pone.0151720.s001.zip › Fig 14 first panel.tif]

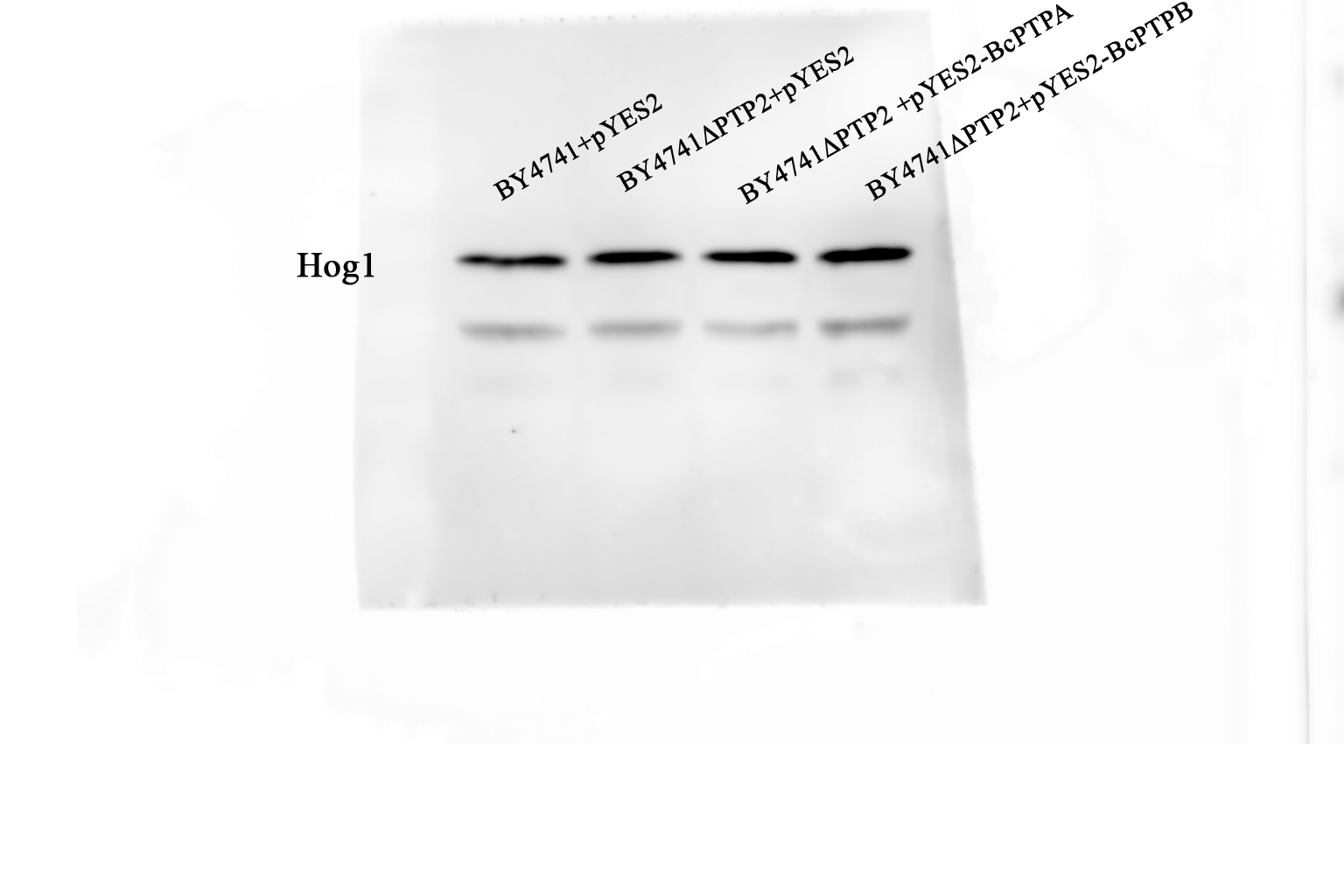

Supplement: S1 File — (ZIP) [file pone.0151720.s001.zip › Fig 14 second panel.tif]

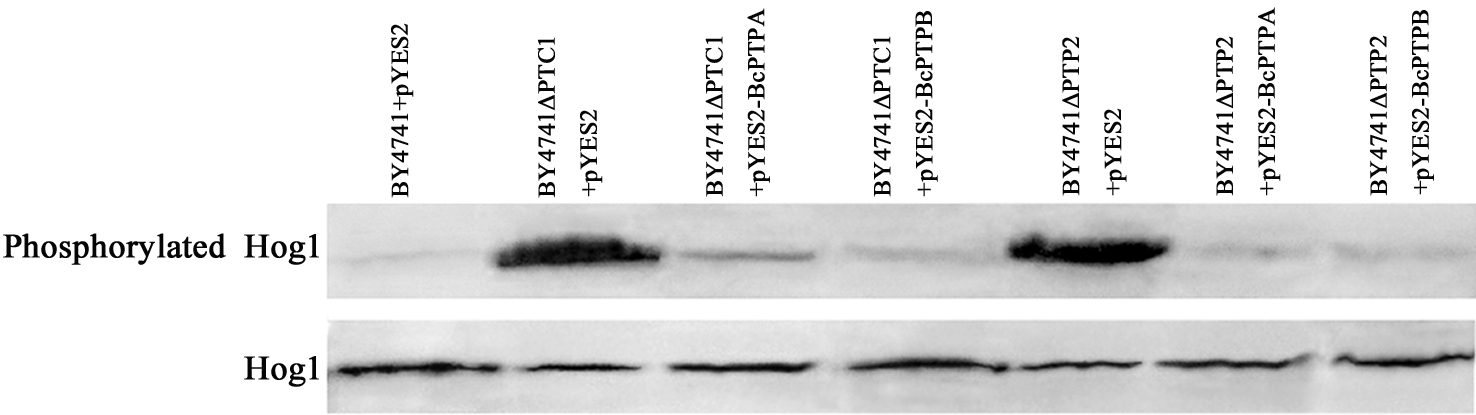

Supplement: S1 Photo — (JPG) [file pone.0151720.s002.jpg]
